# Supplementary material for: Short- and Long-Term Chest-CT Findings after Recovery from COVID-19: A Systematic Review and Meta-Analysis
Source: Diagnostics (Basel). 2024 Mar 14;14(6):621. doi: 10.3390/diagnostics14060621 (PMC10969005; doi:10.3390/diagnostics14060621)
Supplement: Supplementary file 1 [file diagnostics-14-00621-s001.zip › diagnostics-2893222-supplementary.pdf]

Supplemental Materials for Short- and Long-term Chest CT Findings After Recovery from COVID-19: A Systematic Review and Meta-Analysis

Contents

Supplemental Table S1. Breakdown of Comorbidities. 3

Supplemental Table S2. Pooled Event Rates of Chest CT Findings Over Time For All Patients. 11

Supplemental Table S3. Summary of Meta-Regression Models for the Pooled Event Rate Each Lung Abnormality Using Months Since Primary Infection as the Independent Predictor 12

Supplemental Table S4. Pooled Event Rates for Chest CT findings with COVID-19 Severity as the Mediator. 13

Supplemental Table S5. Summary of Meta-Regression Models for the Pooled Event Rate Each Lung Abnormality Stratified by Primary COVID-19 Infection Severity Using Months Since Primary Infection as the Independent Predictor 14

Supplemental Figure S1. Meta Analysis the Follow-up Chest CT Lung Abnormalities Over Time for the Entire Population 15

- Any Abnormality 15
- Fibrotic-like changes 16
- Ground Glass Opacities 17
- Reticulation 18
- Consolidation 19
- Interlobular Septal Thickening 20
- Honeycombing 20

Supplemental Figure S2. Meta-Regression Plots for the Pooled Event Rate Each Lung Abnormality Using Months Since Primary Infection as the Independent Predictor 21

Supplemental Figure S3. Meta Analysis the Follow-up Chest CT Lung Abnormalities Over Time for the Entire Population Stratified by Primary COVID-19 Infection Severity 24

- Any Abnormality 24
  - 1-6 months 24
  - 12-24 Months 25
- Fibrotic-like changes 26
  - 1-6 Months 26
  - 12-24 Months 26
- Ground Glass Opacities 27
  - 1-6 Months 27

12-24 Months 28
Reticulation 29
1-6 Months 29
12-24 Months 30
Consolidation 31
1-6 Months 31
12-24 Months 32
Interlobular Septal Thickening 32
1-6 Months 32
12-24 Months 33
Honeycombing 33
1-6 Months 33
12-24 Months 33
Supplemental Figure S4. Meta-Regression Plots for the Pooled Event Rate Each Lung Abnormality Stratified by Primary COVID-19 Infection Severity Using Months Since Primary Infection as the Independent Predictor 34
Supplemental Figure S5. Risk of Bias Assessment Summary Plot Using Hoy et al. Tool 38
The tool consists of 10 items addressing four domains of bias. Items 1 to 4 assess the external validity of the study (domains are selection and nonresponse bias), and items 5 to 10 assess the internal validity (items 5 to 9 assess the domain of measurement bias, and item 10 assesses bias related to the analysis). 38
Supplemental Figure S6. Funnel Plots for the Pooled Meta-Analysis 39
Supplemental Figure S7. Funnel Plots for the Stratified Meta-Analysis by Severity 42

Supplemental Table S1. Breakdown of Comorbidities.

NR: Not Reported

Author (year)	Comorbidities, n (%)
Bellan et al. (2021)	Arterial hypertension 82 (41)<br>Diabetes 31 (15.5)<br>Dyslipidemia 18 (9)<br>COPD 12 (6)<br>Obesity 22 (11)<br>IBD 4 (2)<br>Chronic liver disease 7 (3.6)<br>Autoimmune disease 3 (1.5)<br>Hematological disease 13 (6.5)<br>Coronary artery disease 18 (9)<br>Atrial fibrillation 13 (6.5)<br>Other structural heart disease 3 (1.5)<br>Other arrhythmogenic heart disease 6 (3)<br>Endocrinological disease 22 (11)<br>CKD 12 (6)<br>Stroke/TIA 5 (2.5)<br>VTE 4 (2)<br>Anxiety and depression 8 (4)<br>Active malignancy 18 (9)
Zhan et al. (2021)	Hypertension 31 (25.6)<br>Diabetes 8 (6.6)<br>Autoimmune diseases 2 (1.7)<br>Cardiovascular diseases 3 (2.5)<br>Cancer 1 (0.8)
Zhou et al. (2021)	Diabetes 6 (5)<br>Hypertension 20 (16.7)<br>Hyperlipidemia 4 (3.3)<br>Coronary heart disease 3 (2.5)
Li et al. (2021)	Hypertension 55 (39.0)<br>Diabetes 52 (36.9)<br>Type 2 diabetes 50 (35.5)<br>Type 1 diabetes 2 (1.4)<br>Cardiovascular disease 13 (9.2)<br>Coronary heart disease 12 (8.5)<br>Heart failure 1 (0.7)<br>Cerebrovascular disease 2 (1.4)

**Author (year)**	**Comorbidities, n (%)**
	Chronic pulmonary disease 14 (9.9)<br>COPD 10 (7.1)<br>Asthma 3 (2.1)<br>Latent Tuberculosis 1 (0.7)<br>Chronic renal failure 1 (0.7)<br>Chronic liver disease 5 (3.5)<br>Chronic hepatitis 2 (1.4)<br>Cirrhosis 3 (2.1)
Chen et al. (2021)	Hypertension 10 (24.4)<br>Type 2 diabetes 4 (9.7)<br>Coronary artery heart disease 2 (4.9)<br>COPD/emphysema 2 (4.9)
Zhao et al. (2021)	Hypertension 16 (17.0)<br>Diabetes Mellitus 9 (9.6)<br>Chronic heart disease 4 (4.3)<br>Asthma 2 (2.1)
Gamberini et al. (2021)	Hypertension 88 (49.4)<br>Chronic ischemic heart disease 13 (7.3)<br>Chronic kidney disease 6 (3.4)<br>COPD 13 (7.3)<br>Diabetes 28 (15.7)
Han et al. (2021)	NR
Wu et al. (2021)	None
Eberst et al. (2022)	Obesity 28 (32.9)<br>Ischemic heart disease 9 (10.6)<br>Heart failure 1 (1.2)<br>Atrial fibrillation 7 (8.2)<br>Stroke 2 (2.4)<br>COPD 7 (8.2)<br>Asthma 6 (7.1)<br>Sleep apnea 16 (18.8)<br>Hypertension 44 (51.8)<br>Diabetes 21 (24.7%) Dyslipidemia 27 (31.8)<br>Deep vein thrombosis 4 (4.7)<br>Pulmonary embolism 0 (0)

**Author (year)**	**Comorbidities, n (%)**
Lorent et al. (2022)	Arterial hypertension 143 (48)<br>Hyperlipidemia 113 (38)<br>Diabetes 75 (25)<br>Ischemic heart disease 36 (12)<br>Active malignancy 57 (19)<br>Chronic lung disease 52 (17)<br>Obstructive sleep apnea syndrome 32 (11)<br>Immune suppression 32 (11)<br>Chronic renal disease 45 (15)<br>Heart failure 15 (5)<br>Transplant (organ/other) 9 (3)
Zangrillo et al. (2021)	NR
Faverio et al. (2022)	Cardiovascular diseases 65 (22.6)<br>Hypertension 82 (28.6)<br>Cerebrovascular diseases 5 (1.7)<br>Asthma 16 (5.6)<br>Obstructive sleep apnea 6 (2.1)<br>Chronic kidney diseases 9 (3.1)<br>Liver diseases 4 (1.4)<br>Diabetes 41 (14.3)<br>Prior cancer 12 (4.2)
Rigoni et al. (2022)	Cardiovascular (including hypertension) 289 (61.5) Diabetes 82 (17.4)<br>Gastrointestinal 73 (15.5)<br>Autoimmune 62 (13.2)<br>Obesity 55 (11.7)<br>Pulmonary 57 (12.1)<br>Renal 50 (10.6)<br>Cancer 56 (11.9)
Liao et al. (2022)	Hypertension 15 (4.9)<br>Hyperlipidemia 4 (1.3)<br>Diabetes 2 (0.7)<br>Heart disease 4 (1.3)<br>Endocrine disease 4 (1.3)<br>Liver disease 4 (1.3)<br>Solid tumor 2 (0.7)
González et al. (2022)	Obesity 81 (45.5)<br>Hypertension 78 (43.1)

**Author (year)**	**Comorbidities, n (%)**
	Diabetes mellitus (Type I/II) 42 (23.2)<br>Chronic heart disease 22 (12.2)<br>COPD/Bronchiectasis 14 (7.7)<br>Chronic renal disease 11 (6.1)<br>Asthma 10 (5.5)<br>HIV 2 (1.1)<br>Immunological disorders 1 (0.6)
Corsi et al. (2022)	Arterial hypertension 42 (66.8)<br>Cardiovascular disease 14 (22.2)<br>Obesity 20 (31.7)<br>Diabetes 12 (19)<br>Dyslipidemia 24 (38.1)<br>Chronic renal failure 1 (1.6)<br>Neoplasia (active history) 1 (1.6)<br>Rheumatic pathology 5 (7.9)<br>Immunodepression 3 (4.8)<br>Epilepsy 1 (1.6)
Zhang et al. (2022)	NR
Liu et al. 2022	Hypertension 222 (37.4)<br>Diabetes 103 (17.3)<br>Cardiovascular disease 37 (6.2)<br>Chronic lung diseases 50 (8.4)<br>Malignancy 20 (3.4)<br>Cerebrovascular disease 15 (2.5)<br>Chronic hepatitis B 17 (2.9)<br>Chronic kidney disease 2 (0.3)<br>Gastrointestinal diseases 10 (1.7)<br>Metabolic arthritis 7 (1.2)<br>Autoimmune disease 6 (1.0)
Marando et al. 2022	Hypertension 14 (36.8)<br>Diabetes 5 (13.1)<br>Cardiovascular diseases 7 (18.4)<br>Coronary heart disease 4 (10.5)<br>Chronic respiratory diseases 8 (21)<br>COPD 3 (7.9)<br>Asthma 5 (13.1)<br>Chronic kidney disease 3 (7.9)<br>Malignancy 4 (10.5)

Author (year)	Comorbidities, n (%)
	Depression 4 (10.5)
Luger et al. 2022	NR
Pan et al. 2022	Hypertension 28 (13.4)<br>Type 2 diabetes mellitus 13 (6.2)<br>Coronary heart disease 11 (5.3)
Tarraso et al. 2022	Pulmonary disease 39 (18.7)<br>Hypertension 82 (39.2)<br>Diabetes 30 (14.4)<br>Cardiovascular disease 21 (10.0)
Vijayakumar et al. 2022	Asthma 6 (18.8)<br>COPD 2 (6.3)<br>Hypertension 11 (34.4)<br>Ischemic heart disease 3 (9.4)<br>Hypercholesterolemia 4 (12.5)<br>Type 2 diabetes mellitus 8 (25)<br>Previous venous thromboembolism 1 (3.1)<br>Anxiety and/or depression 4 (12.5)
Martino et al. 2022	Hypertension 33 (51.5)<br>Diabetes 12 (18.7)<br>Obesity 8 (12.5)<br>Heart disease 16 (25)<br>Lung disease 11 (17.2)<br>Active cancer 4 (6.2)<br>Rheumatic diseases 6 (9.4)
Bocchino et al. 2022	Type II diabetes 23 (27.4)<br>Arterial systemic hypertension 55 (65.5)<br>Cardiovascular diseases 17 (20.2)<br>Chronic lung diseases 7 (8.3)<br>Obesity 24 (28.6)
Huang et al. 2022	Hypertension 410 (34.4)<br>Diabetes 164 (13.8)<br>Coronary heart diseases 104 (8.7)<br>Cerebrovascular diseases 66 (5.5)<br>Chronic kidney disease 50 (4.2)<br>Malignancy 31 (2.6)

Author (year)	Comorbidities, n (%)
	COPD 18 (1.5)
Barini et al. 2022	NR
van Raaij et al. 2022	Hypertension 18 (27.3)<br>Diabetes mellitus 14 (21.2)<br>Asthma 9 (13.6)<br>Coronary artery disease 7 (10.6)<br>Hypercholesterolemia 6 (9.1)<br>Immunodeficiency 4 (6.1)<br>Heart valve anomaly 4 (6.1)<br>Chronic kidney disease 4 (6.1)<br>Atrial fibrillation or flutter 3 (4.5)<br>Cerebrovascular diseases 2 (3)<br>Chronic obstructive pulmonary disease 2 (3)<br>Pulmonary embolism (before COVID-19) 1 (1.5)
Lenoir et al. 2022	Interstitial lung disease 8 (2)<br>Asthma 74 (15)<br>Coronary artery disease 46 (9)<br>Arterial hypertension 207 (40)<br>Pulmonary hypertension 9 (2)<br>Heart failure 29 (6)<br>Pulmonary embolism/DVT 20 (4)<br>GERD with PPI 45 (9)<br>GERD without PPI 11 (2)<br>Sleep apnea 68 (14)<br>Lung cancer 4 (1)<br>Other cancer 46 (9)<br>Connective tissue disease or vasculitis 4 (1)<br>Depression/anxiety 49 (11)<br>Diabetes 94 (18)<br>Chronic renal failure 31 (7)<br>Solid organ transplant 5 (1)<br>Other comorbidities 224 (47)
Guo et al. 2022	Hypertension 75 (36.1)<br>Diabetes 23 (11.1)<br>Cardiovascular disease 14 (6.7)
Bernardinello et al. 2023	Cardiovascular diseases 174 (50.1)<br>Respiratory diseases 50 (14.5)

Author (year)	Comorbidities, n (%)
	Autoimmune diseases 52 (15)<br>Metabolic diseases 158 (45.5)<br>Oncologic diseases 57 (16.4)
Han et al. 2023	Type 2 diabetes15 (10)<br>Hypertension60 (42)<br>Cardiovascular disease7 (5)
Bongiovanni et al. 2023	COPD 31 (13.3)<br>Asthma 26 (11.2)<br>Diabetes 76 (32.6)<br>Cardiovascular 119 (51.1)<br>Autoimmune 19 (8.2)<br>Renal failure 21 (9)
Lerum et al. 2023	Diabetes mellitus 22 (8)<br>Hypertension 80 (33)<br>COPD 13 (5)<br>Obesity 78 (31)
Sanna et al. 2023	Arterial hypertension 43 (43)<br>COPD 7 (7)<br>Asthma 14 (14)<br>Interstitial lung disease 0 (0)<br>Obesity/overweight 64 (64)
Núñez-Fernández et al. 2023	Chronic cardiopathy 36 (18.6)<br>Diabetes 21 (10.8)<br>Hypertension 71 (36.6)<br>COPD 13 (6.7)<br>Chronic kidney failure 6 (3.1)
Mulet et al. 2023	Pulmonary disease 23 (17)<br>Hypertension 47 (34.8)<br>Diabetes 28 (20.7)<br>Cardiovascular disease 16 (11.9)
Noureddine et al. 2023	Obesity (BMI>30) 33 (55)<br>Ischemic heart disease 5 (8.3)<br>Hypertension 28 (46.7)<br>Dyslipidemia 21 (35)<br>Diabetes 15 (25)

Author (year)	Comorbidities, n (%)
	COPD 5 (8.3)<br>Asthma 6 (10)<br>Sleep apnea 12 (20)<br>Deep vein thrombosis 3 (5)<br>Pulmonary embolism 0 (0)
Sahanic et al. 2023	Metabolic disease 42 (45)<br>Diabetes 15 (16)<br>Hypercholesterolemia 21 (23)<br>Cardiovascular disease 40 (43)<br>Pulmonary disease 19 (20)<br>Malignancy 9.3 (10)<br>Immune deficiency 5.6 (6)<br>Chronic kidney disease 6.5 (7)<br>Gastrointestinal disease 13 (14)
van der Sar-van der Brugge et al. 2023	Diabetes mellitus 30 (19)<br>Cardiovascular disease 41 (25)<br>COPD 25 (16)<br>Asthma 23 (14.3)<br>Chronic kidney disease 11 (7)<br>Malignancy 18 (11)<br>Chronic hematologic disease 3 (2)
Schlemmer et al., 2023	Cardiovascular disease 244 (50.3)<br>COPD 12 (2.5)<br>Emphysema 17 (3.5)<br>Asthma 32 (6.6)<br>Interstitial lung disease 8 (1.6)<br>Non-cystic fibrosis bronchiectasis 5 (1.0)<br>Obstructive sleep apnea 46 (9.5)<br>Diabetes 107 (22.1)<br>Immune deficiency (all causes) 52 (10.7)
Flor et al., 2023	NR

Supplemental Table S2. Pooled Event Rates of Chest CT Findings Over Time For All Patients.

UE: Unable to estimate. *$p < 0.05$

	1-6 months		12-24 months	
**Chest CT findings**	**Event rate (95% CI)**	**I^2**	**Event rate (95% CI)**	**I^2**
Any Abnormality	0.75 (0.63-0.84)*	0.89	0.63 (0.49-0.75)*	0.95
Bronchiectasis	0.18 (0.11-0.29)*	0.95	0.17 (0.10-0.28)*	0.95
Consolidation	0.06 (0.03-0.09)*	0.93	0.03 (0.02-0.04)*	0.70
Fibrotic-like Changes	0.44 (0.3-0.59)*	0.9	0.38 (0.23-0.56)*	0.94
GGO	0.43 (0.32-0.55)*	0.94	0.25 (0.17-0.35)*	0.93
Honeycombing	0.03 (0.02-0.07)	UE	0.04 (0.02-0.07)	0.40
Reticulation	0.29 (0.17-0.45)*	0.93	0.28 (0.18-0.41)*	0.92
Interlobular Septal Thickening	0.25 (0.13-0.44)*	0.94	0.22 (0.06-0.55)*	0.92

Supplemental Table S3. Summary of Meta-Regression Models for the Pooled Event Rate Each Lung Abnormality Using Months Since Primary Infection as the Independent Predictor

Abnormality	Beta Coefficient (Months)	P-Value
Any Abnormality	-0.137	0.002
GGO	-0.169	<0.001
Fibrotic-Like Changes	0.047	0.265
Bronchiectasis	0.034	0.373
Reticulation	-0.002	0.955
Consolidation	-0.177	<0.001
Interlobular Septal Thickening	-0.118	0.096
Honeycombing	0.075	0.03

Supplemental Table S4. Pooled Event Rates for Chest CT findings with COVID-19 Severity as the Mediator.

UE: Unable to estimate. *p<0.05, **p≤0.01

Chest CT Findings	Severe subgroup				Non-severe subgroup				Non-severe vs. severe	
	1-6 months		12-24 months		1-6 months		12-24 months			
	Event rate (95%CI)	I^2	Event rate (95%CI)	I^2	Event rate (95%CI)	I^2	Event rate (95%CI)	I^2	P (1-6 months)	P (12-24 months)
Any Abnormality	0.6 (0.39-0.78)	0.77	0.39 (0.21-0.6)	0.86	0.88 (0.75-0.95)**	0.92	0.75 (0.63-0.84)**	0.94	0.01	<0.001
Bronchiectasis	0.09 (0.06-0.14)**	0.56	0.09 (0.04-0.16)**	0.71	0.29 (0.16-0.46)**	0.96	0.24 (0.13-0.4)**	0.96	<0.001	0.02
Consolidation	0.04 (0.02-0.09)**	0	0.01 (0-0.05)**	0.64	0.1 (0.06-0.17)**	0.93	0.05 (0.03-0.07)**	0.61	0.06	0.07
Fibrotic-like Changes	0.35 (0.26-0.46)**	0.78	0.21 (0.11-0.34)**	0.93	0.5 (0.28-0.72)*	0.83	0.45 (0.27-0.65)**	0.92	0.27	0.03
GGO	0.34 (0.17-0.57)	0.9	0.17 (0.1-0.28)**	0.87	0.44 (0.29-0.6)*	0.93	0.26 (0.17-0.38)	0.88	0.50	0.21
Honeycombing	0 (0-1)	UE	UE	UE	0.06 (0.04-0.09)**	0	0.05 (0.03-0.09)**	0.60	1	UE
Reticulation	0.12 (0.06-0.23)**	0.84	0.07 (0.02-0.17)**	0.72	0.36 (0.2-0.56)*	0.93	0.33 (0.21-0.49)*	0.92	0.01	<0.001
Interlobular Septal Thickening	0.11 (0.08-0.16)**	0.53	0.07 (0.05-0.1)**	0	0.28 (0.1-0.57)	0.94	0.22 (0.05-0.63)	0.81	0.09	0.16

Supplemental Table S5. Summary of Meta-Regression Models for the Pooled Event Rate Each Lung Abnormality Stratified by Primary COVID-19 Infection Severity Using Months Since Primary Infection as the Independent Predictor

**Abnormality**	Non-Severe		Severe	
	**Beta Coefficient (Months)**	**P-Value**	**Beta Coefficient (Months)**	**P-Value**
Any Abnormality	-0.192	0.01	-0.11	0.032
GGO	-0.181	0.001	-0.148	<0.001
Fibrotic-Like Changes	0.016	0.793	0.087	0.109
Bronchiectasis	0.03	0.595	-0.006	0.901
Reticulation	-0.058	0.369	-0.04	0.324
Consolidation	-0.278	0.001	-0.145	0.001
Interlobular Septal Thickening	-0.069	0.004	-0.136	0.094
Honeycombing	Not Enough Effect Sizes		0.033	0.377

Supplemental Figure S1. Meta Analysis the Follow-up Chest CT Lung Abnormalities Over Time for the Entire Population

Any Abnormality

Study	Events	Total	Proportion	95%-CI
**Follow-up Period = 1-6 months**				
Bocchino, 2022	50	84	0.60	[0.48; 0.70]
Bongiovanni, 2023	149	233	0.64	[0.57; 0.70]
Corsi, 2022	49	63	0.78	[0.66; 0.87]
Eberst, 2022	77	80	0.96	[0.89; 0.99]
Flor, 2023	18	18	1.00	[0.81; 1.00]
Han, 2021	62	62	1.00	[0.94; 1.00]
Han, 2023	78	144	0.54	[0.46; 0.62]
Huang, 2022	128	128	1.00	[0.97; 1.00]
Li, 2021	77	141	0.55	[0.46; 0.63]
Lorent, 2022	158	297	0.53	[0.47; 0.59]
Luger, 2022	47	70	0.67	[0.55; 0.78]
Marando, 2022	32	39	0.82	[0.66; 0.92]
Martino, 2022	42	52	0.81	[0.67; 0.90]
Mulet, 2023	72	135	0.53	[0.45; 0.62]
Pan, 2022	81	209	0.39	[0.32; 0.46]
Rigoni, 2022	65	105	0.62	[0.52; 0.71]
Sahanic, 2023	45	78	0.58	[0.46; 0.69]
Sanna, 2023	78	100	0.78	[0.69; 0.86]
Schlemmer, 2023	196	225	0.87	[0.82; 0.91]
Tarraso, 2022	200	325	0.62	[0.56; 0.67]
Vijayakumar, 2022	41	80	0.51	[0.40; 0.63]
Wu, 2021	40	83	0.48	[0.37; 0.59]
Zhan, 2021	21	62	0.34	[0.22; 0.47]
van Raaij, 2022	54	64	0.84	[0.73; 0.92]
**Random effects model**		2877	0.75	[0.63; 0.84]
Heterogeneity: $I^2 = 89\%$, $\tau^2 = 1.8621$, $p < 0.01$				
**Follow-up Period = 12-24 months**				
Bellan, 2021	44	190	0.23	[0.17; 0.30]
Bernardinello, 2023	24	347	0.07	[0.04; 0.10]
Bocchino, 2022	6	84	0.07	[0.03; 0.15]
Bongiovanni, 2023	140	233	0.60	[0.53; 0.66]
Chen, 2021	17	36	0.47	[0.30; 0.65]
Corsi, 2022	48	63	0.76	[0.64; 0.86]
Eberst, 2022	60	64	0.94	[0.85; 0.98]
Faverio, 2021	178	270	0.66	[0.60; 0.72]
Flor, 2023	18	18	1.00	[0.81; 1.00]
González, 2022	41	41	1.00	[0.91; 1.00]
Han, 2021	45	62	0.73	[0.60; 0.83]
Han, 2023	56	144	0.39	[0.31; 0.47]
Huang, 2022	47	57	0.82	[0.70; 0.91]
Li, 2021	13	25	0.52	[0.31; 0.72]
Liao, 2022	96	256	0.38	[0.32; 0.44]
Lorent, 2022	68	105	0.65	[0.55; 0.74]
Luger, 2022	49	91	0.54	[0.43; 0.64]
Marando, 2022	30	31	0.97	[0.83; 1.00]
Martino, 2022	30	47	0.64	[0.49; 0.77]
Mulet, 2023	46	125	0.37	[0.28; 0.46]
Noureddine, 2023	50	60	0.83	[0.71; 0.92]
Pan, 2022	53	209	0.25	[0.20; 0.32]
Sahanic, 2023	52	101	0.51	[0.41; 0.62]
Sanna, 2023	19	19	1.00	[0.82; 1.00]
Schlemmer, 2023	114	123	0.93	[0.87; 0.97]
Tarraso, 2022	123	156	0.79	[0.72; 0.85]
Vijayakumar, 2022	27	32	0.84	[0.67; 0.95]
Wu, 2021	20	83	0.24	[0.15; 0.35]
Zhan, 2021	10	121	0.08	[0.04; 0.15]
Zhang, 2022	114	199	0.57	[0.50; 0.64]
Zhao, 2021	67	94	0.71	[0.61; 0.80]
Zhou, 2021	55	97	0.57	[0.46; 0.67]
van Raaij, 2022	34	64	0.53	[0.40; 0.66]
van der Sar-van der Brugge, 2023	33	66	0.50	[0.37; 0.63]
**Random effects model**		3713	0.63	[0.49; 0.75]
Heterogeneity: $I^2 = 95\%$, $\tau^2 = 2.5779$, $p < 0.01$				

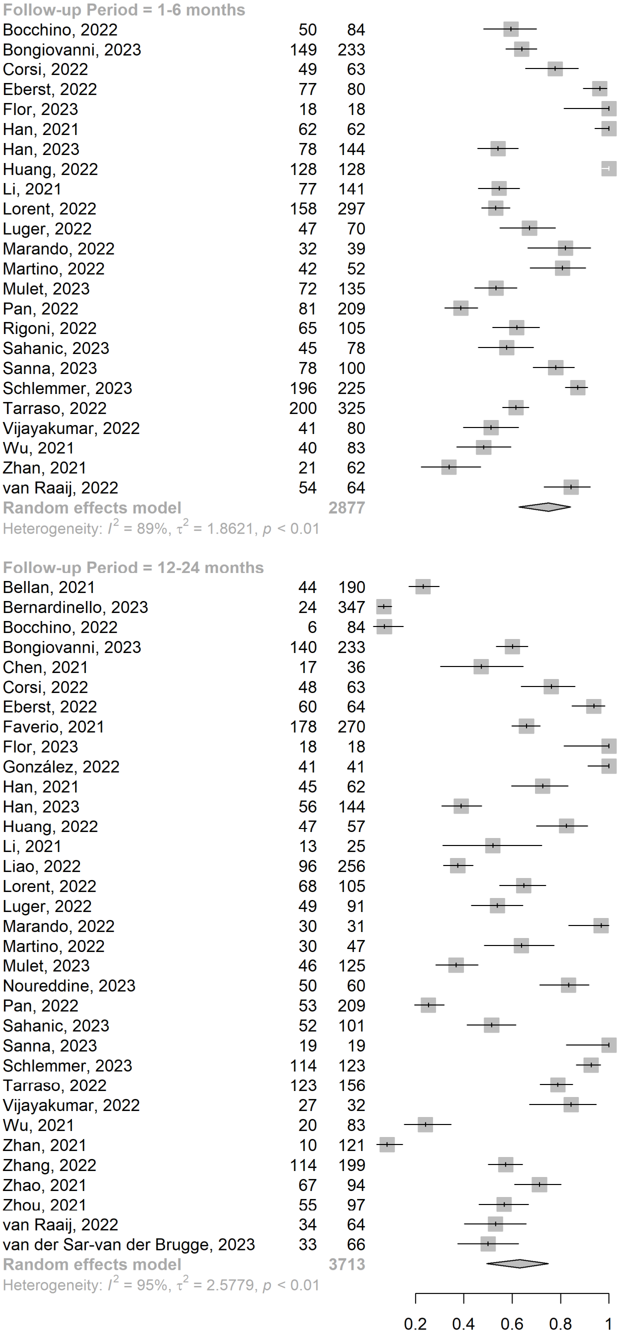

Fibrotic-like changes

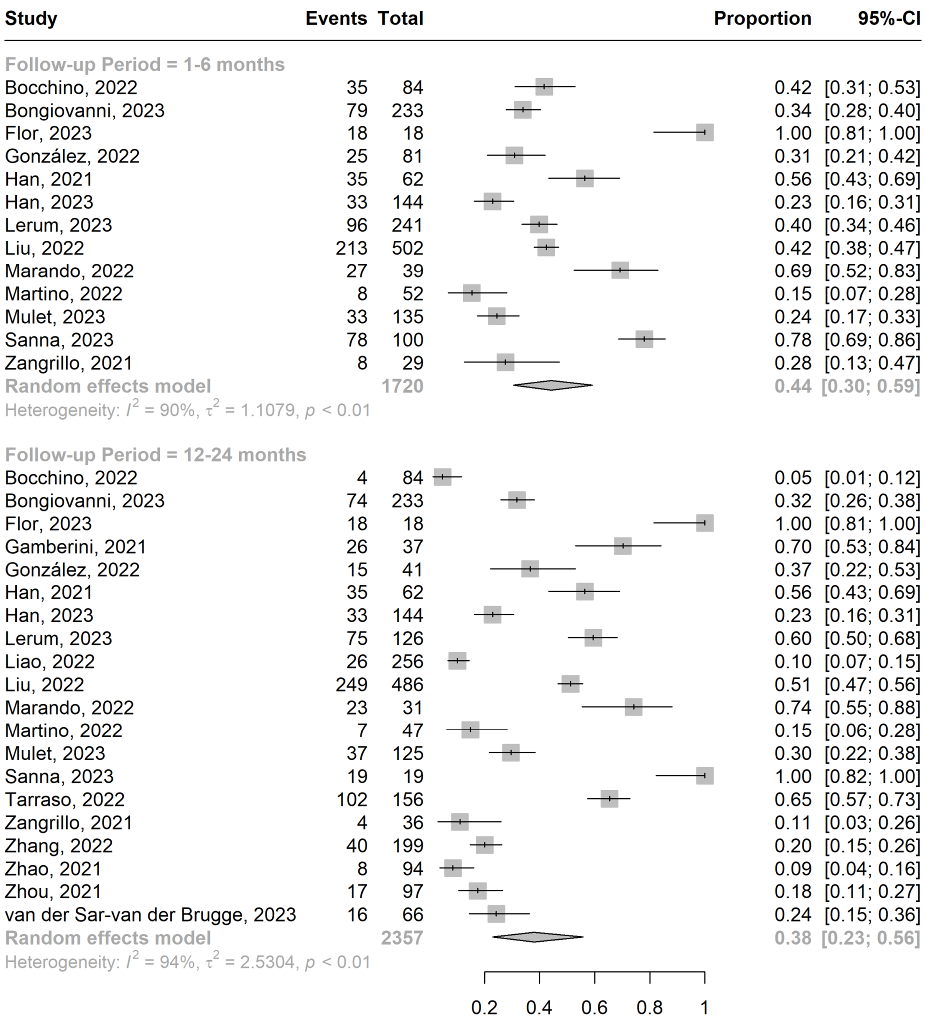

Ground Glass Opacities

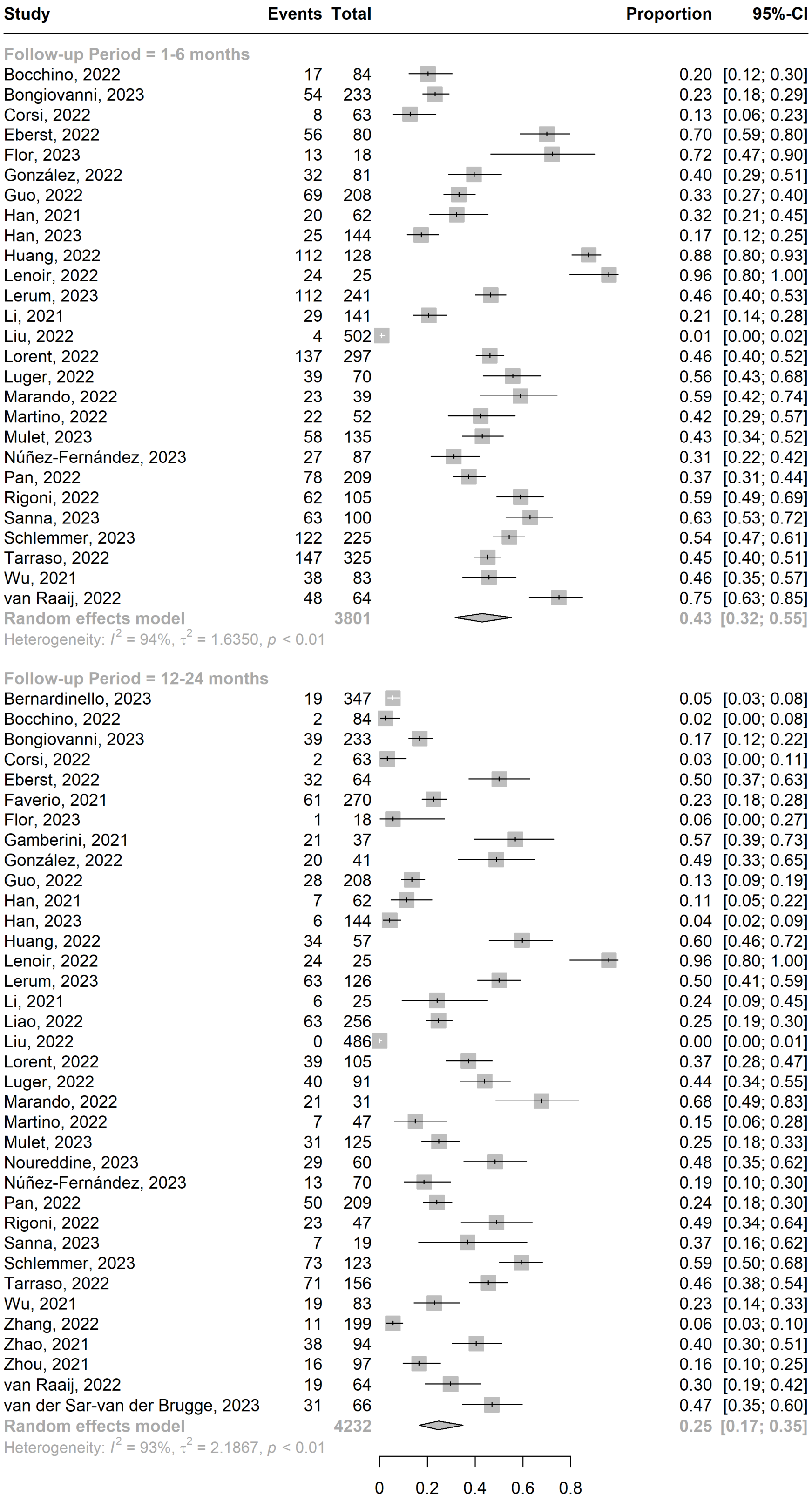

Reticulation

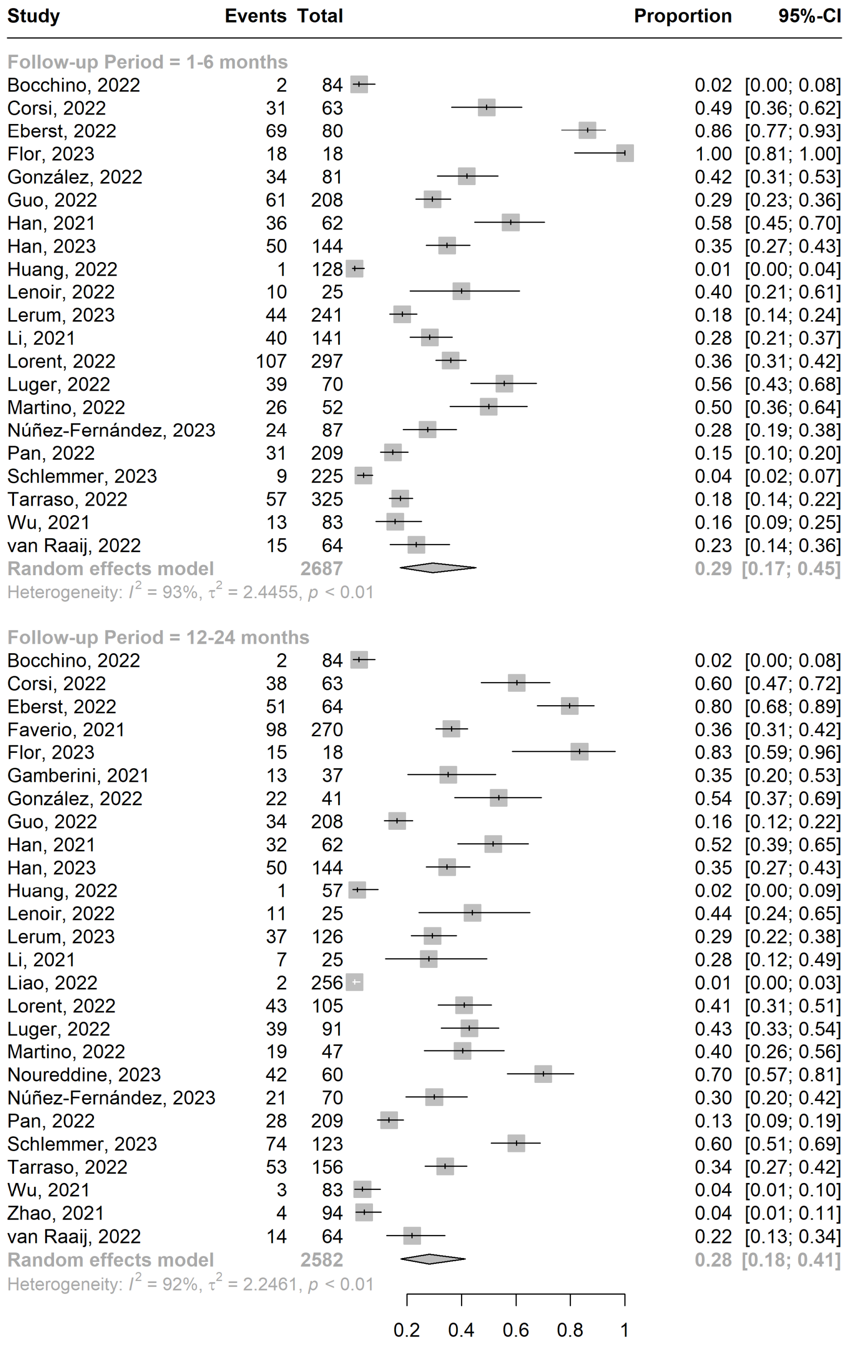

Consolidation

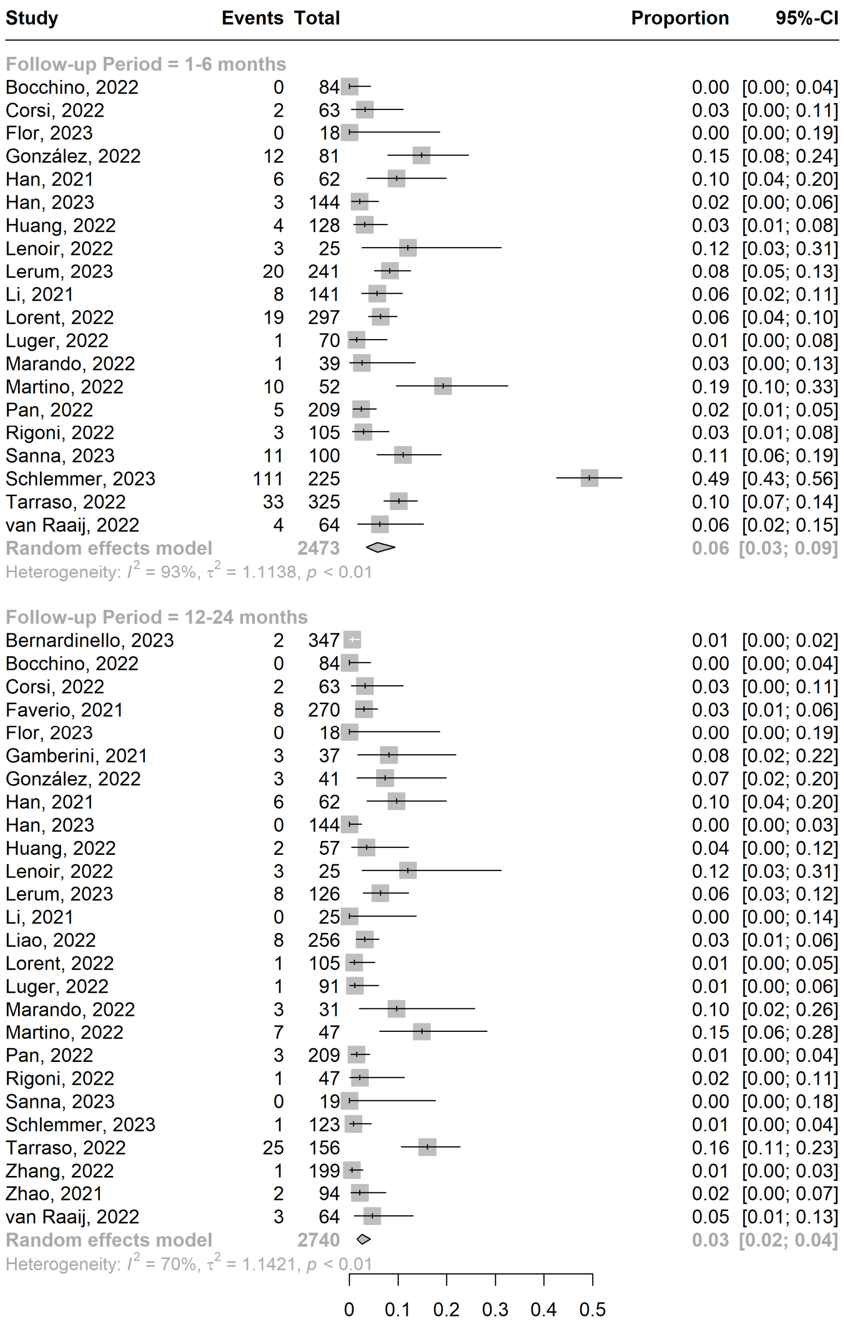

Interlobular Septal Thickening

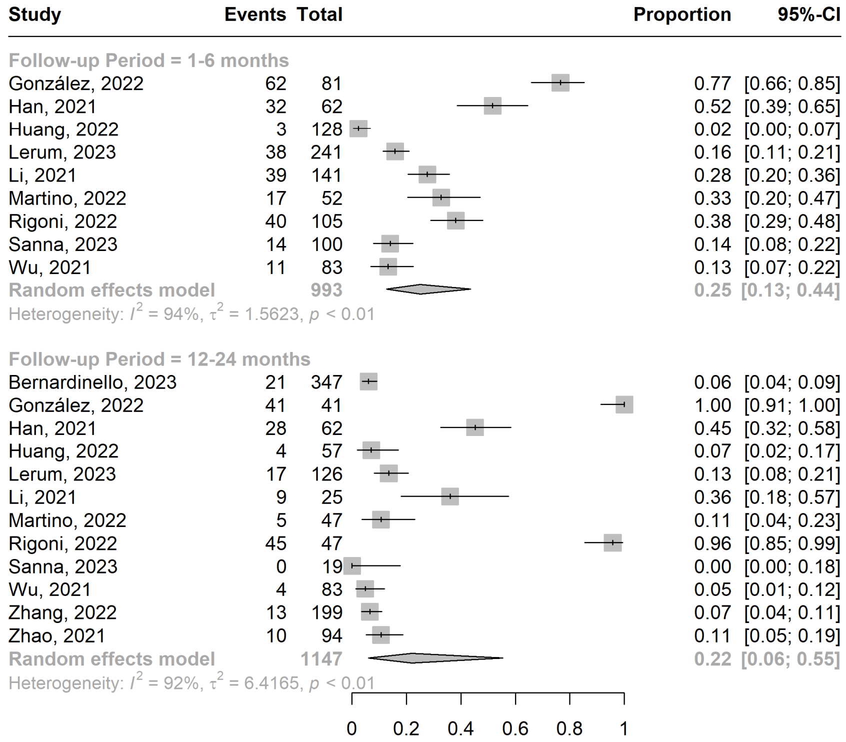

Honeycombing

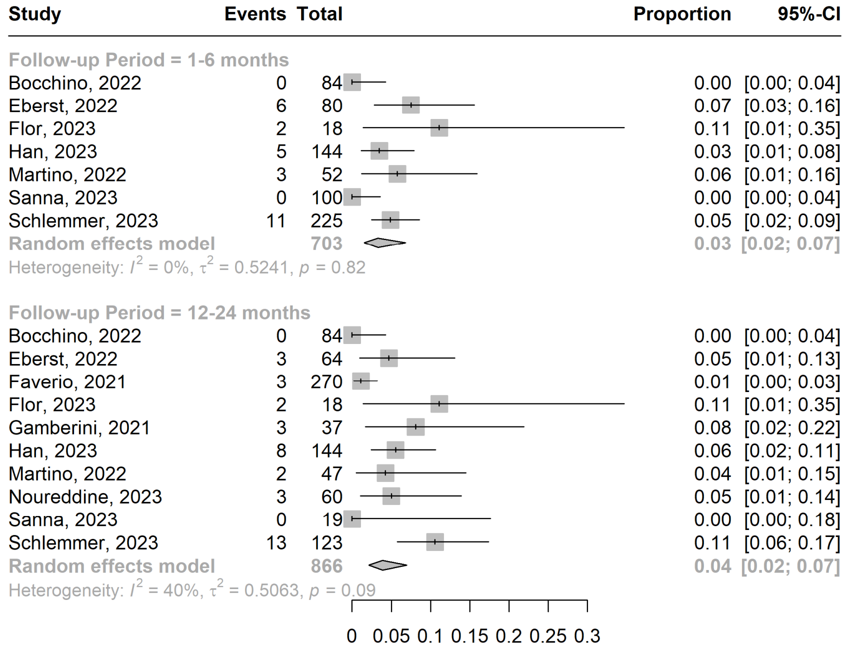

Supplemental Figure S2. Meta-Regression Plots for the Pooled Event Rate Each Lung Abnormality Using Months Since Primary Infection as the Independent Predictor

Any Abnormality

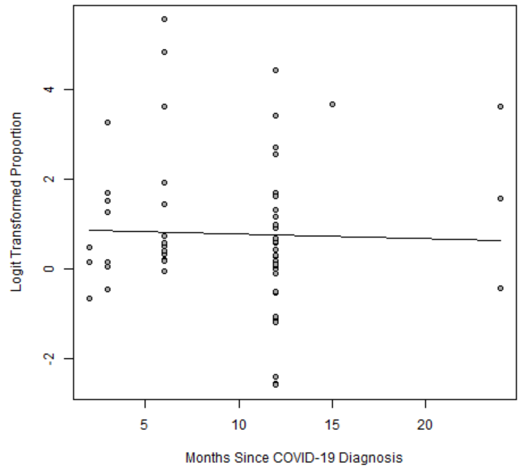

Bronchiectasis

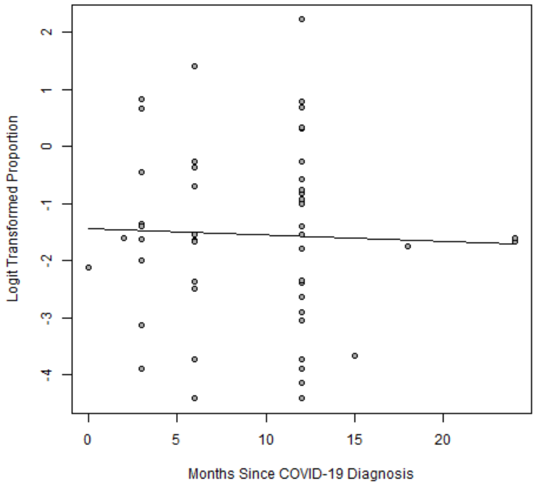

Consolidation

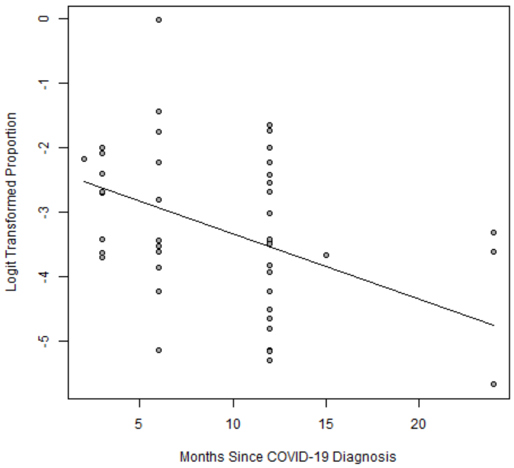

Fibrotic Like Changes

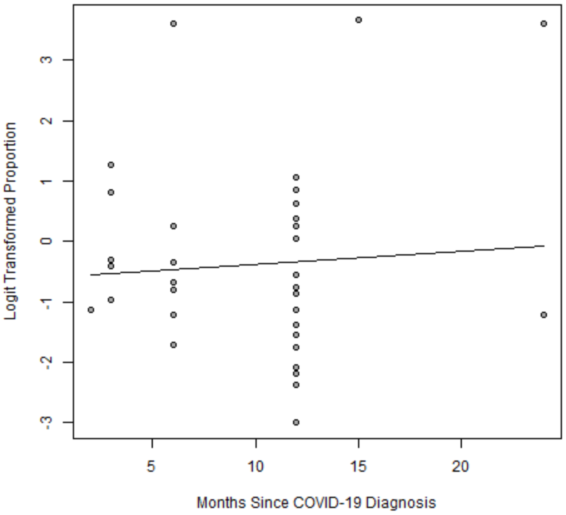

GGO

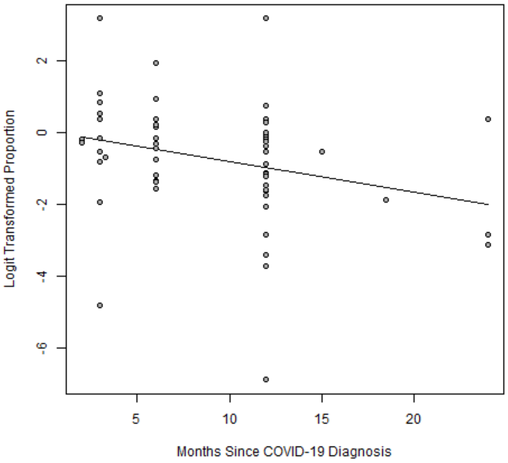

Honeycombing

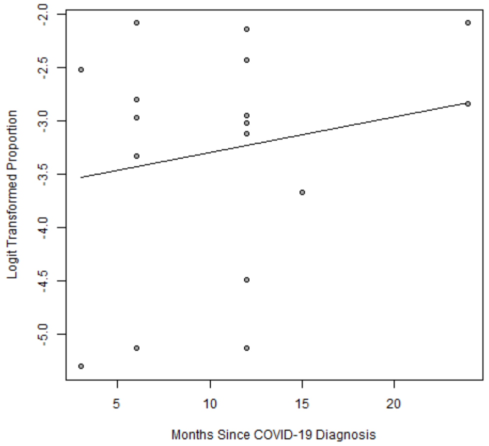

Interlobular Septal Thickening

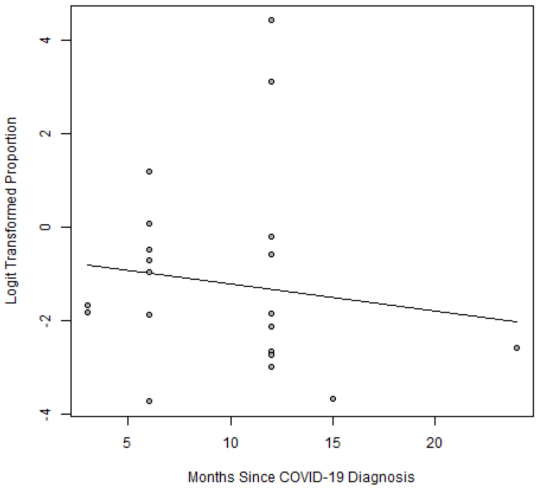

Reticulation

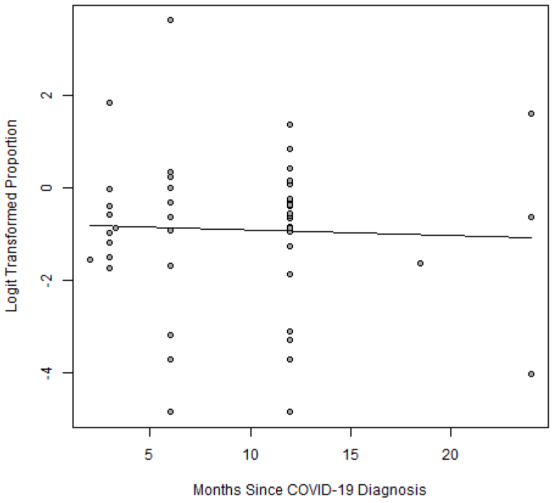

Supplemental Figure S3. Meta Analysis the Follow-up Chest CT Lung Abnormalities Over Time for the Entire Population Stratified by Primary COVID-19 Infection Severity

Any Abnormality

1-6 months

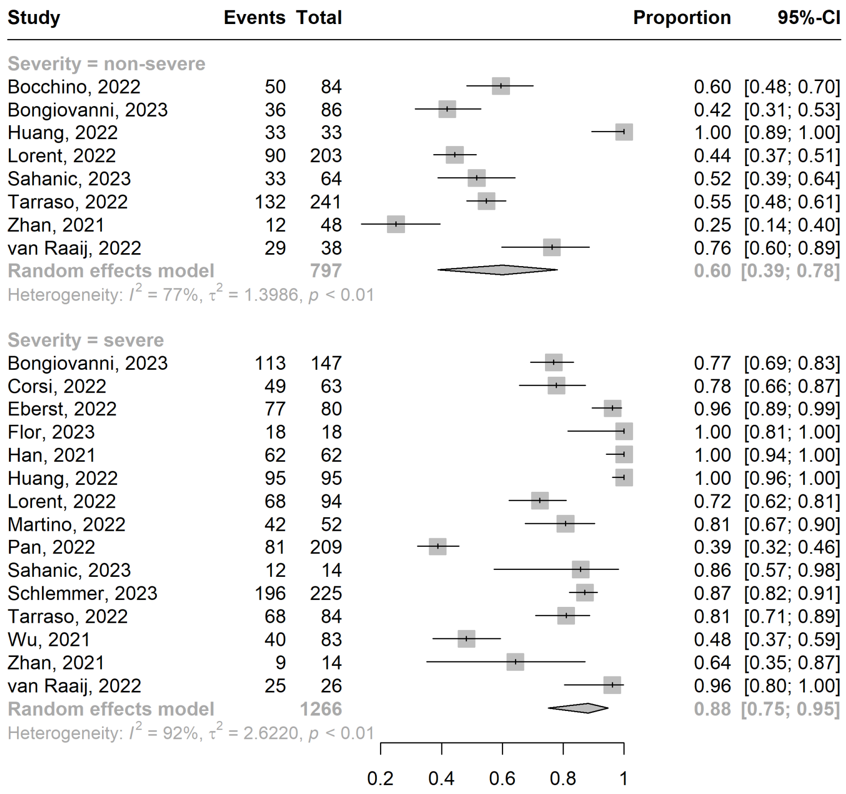

12-24 Months

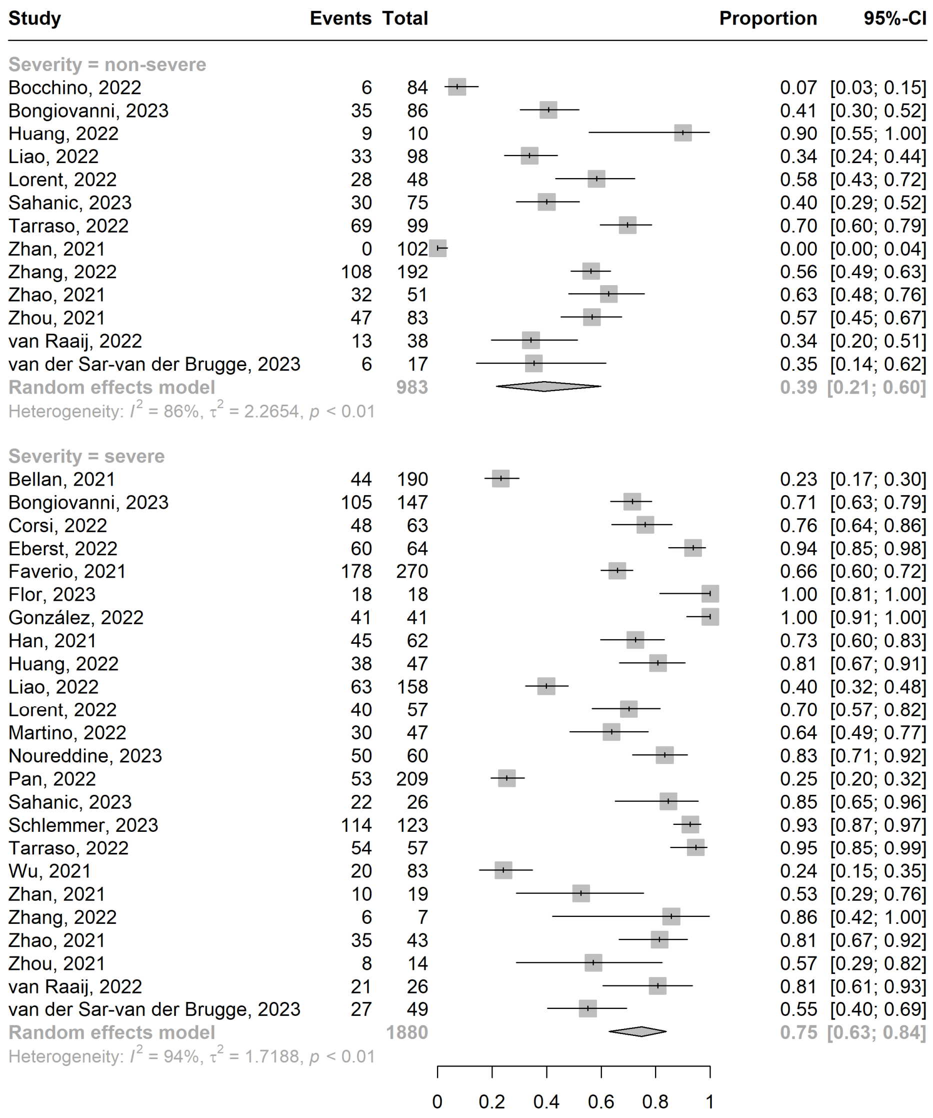
Study Events Total Proportion 95%-CI
Severity = non-severe
Bocchino, 2022 6 84 0.07 [0.03; 0.15]
Bongiovanni, 2023 35 86 0.41 [0.30; 0.52]
Huang, 2022 9 10 0.90 [0.55; 1.00]
Liao, 2022 33 98 0.34 [0.24; 0.44]
Lorent, 2022 28 48 0.58 [0.43; 0.72]
Sahanic, 2023 30 75 0.40 [0.29; 0.52]
Tarraso, 2022 69 99 0.70 [0.60; 0.79]
Zhan, 2021 0 102 0.00 [0.00; 0.04]
Zhang, 2022 108 192 0.56 [0.49; 0.63]
Zhao, 2021 32 51 0.63 [0.48; 0.76]
Zhou, 2021 47 83 0.57 [0.45; 0.67]
van Raaij, 2022 13 38 0.34 [0.20; 0.51]
van der Sar-van der Brugge, 2023 6 17 0.35 [0.14; 0.62]
Random effects model 983 0.39 [0.21; 0.60]
Heterogeneity: I^2 = 86%, τ^2 = 2.2654, p < 0.01
Severity = severe
Bellan, 2021 44 190 0.23 [0.17; 0.30]
Bongiovanni, 2023 105 147 0.71 [0.63; 0.79]
Corsi, 2022 48 63 0.76 [0.64; 0.86]
Eberst, 2022 60 64 0.94 [0.85; 0.98]
Faverio, 2021 178 270 0.66 [0.60; 0.72]
Flor, 2023 18 18 1.00 [0.81; 1.00]
González, 2022 41 41 1.00 [0.91; 1.00]
Han, 2021 45 62 0.73 [0.60; 0.83]
Huang, 2022 38 47 0.81 [0.67; 0.91]
Liao, 2022 63 158 0.40 [0.32; 0.48]
Lorent, 2022 40 57 0.70 [0.57; 0.82]
Martino, 2022 30 47 0.64 [0.49; 0.77]
Noureddine, 2023 50 60 0.83 [0.71; 0.92]
Pan, 2022 53 209 0.25 [0.20; 0.32]
Sahanic, 2023 22 26 0.85 [0.65; 0.96]
Schlemmer, 2023 114 123 0.93 [0.87; 0.97]
Tarraso, 2022 54 57 0.95 [0.85; 0.99]
Wu, 2021 20 83 0.24 [0.15; 0.35]
Zhan, 2021 10 19 0.53 [0.29; 0.76]
Zhang, 2022 6 7 0.86 [0.42; 1.00]
Zhao, 2021 35 43 0.81 [0.67; 0.92]
Zhou, 2021 8 14 0.57 [0.29; 0.82]
van Raaij, 2022 21 26 0.81 [0.61; 0.93]
van der Sar-van der Brugge, 2023 27 49 0.55 [0.40; 0.69]
Random effects model 1880 0.75 [0.63; 0.84]
Heterogeneity: I^2 = 94%, τ^2 = 1.7188, p < 0.01
0 0.2 0.4 0.6 0.8 1

Fibrotic-like changes

1-6 Months

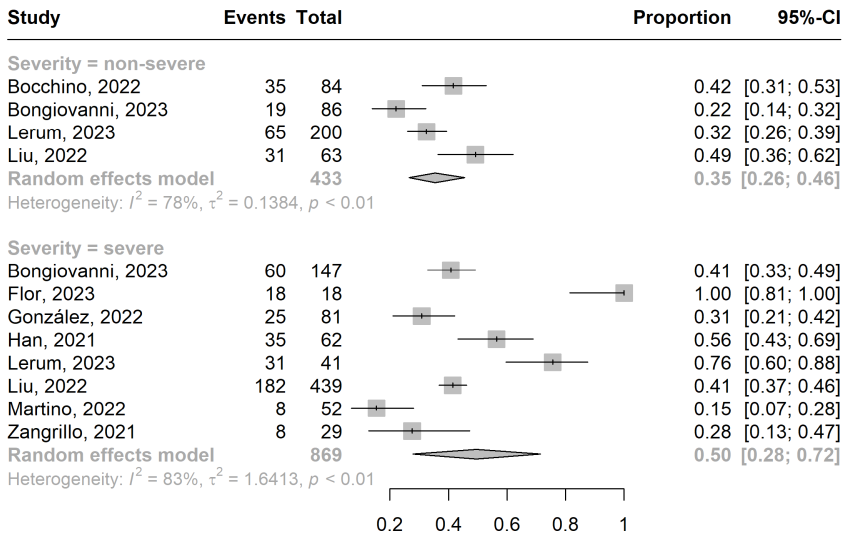

12-24 Months

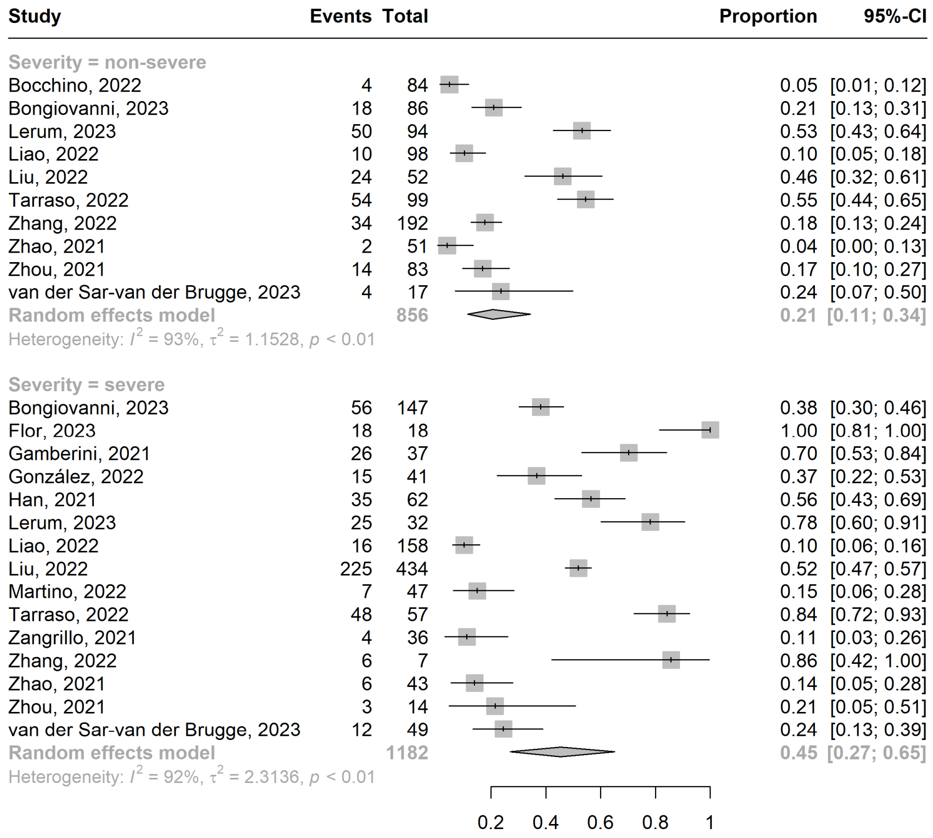

Ground Glass Opacities

1-6 Months

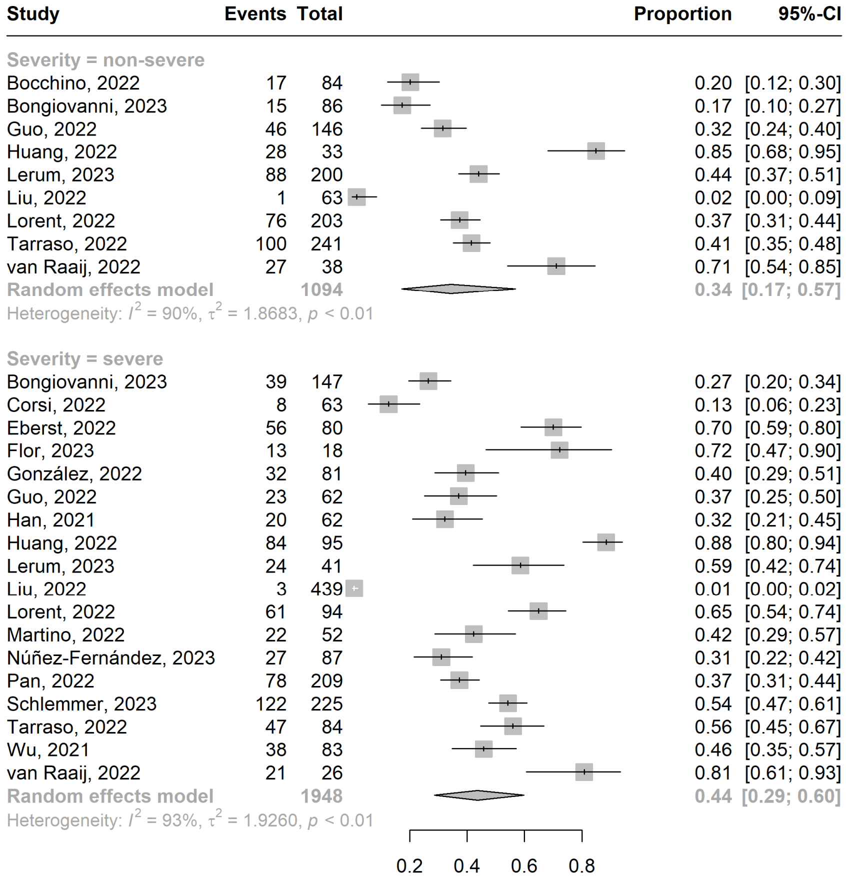

12-24 Months

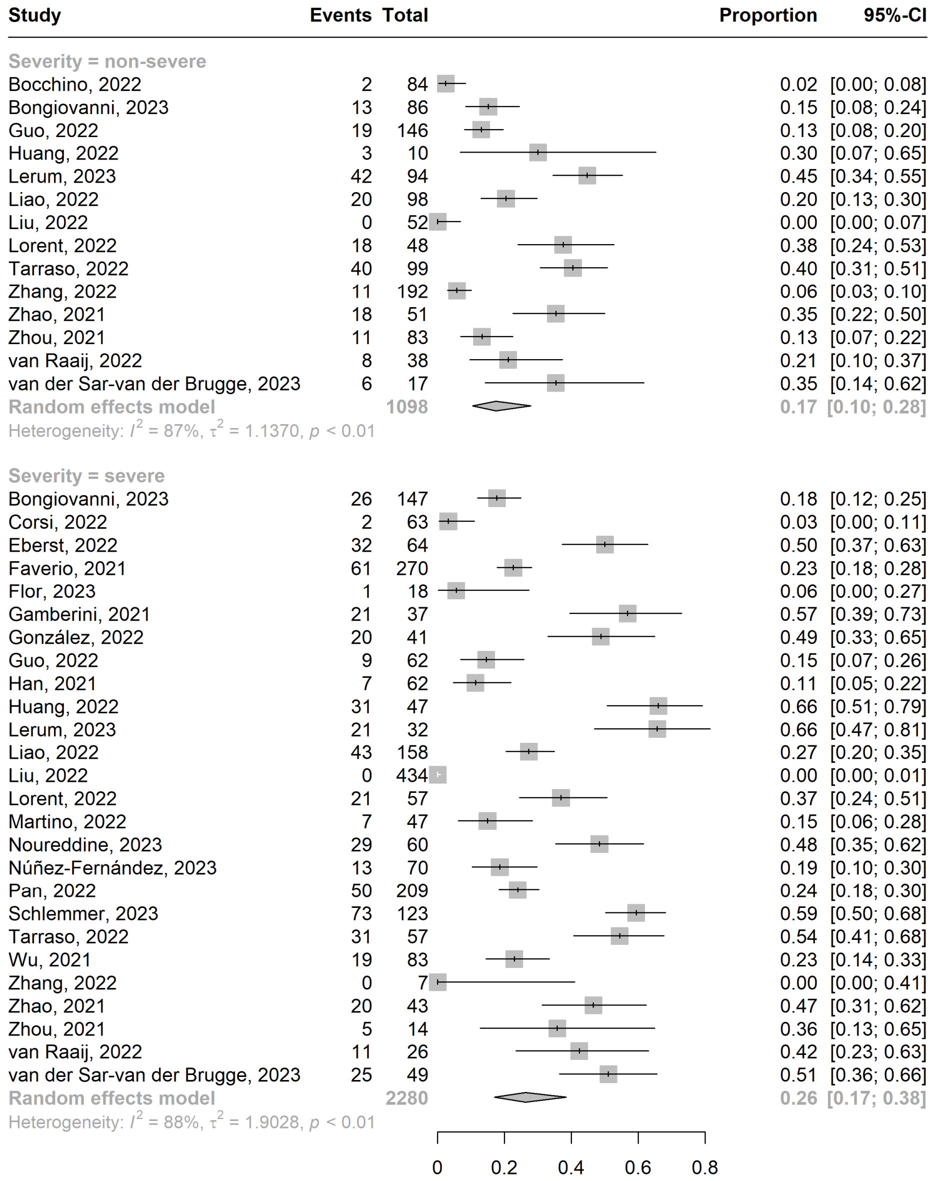
Study Events Total Proportion 95%-CI
Severity = non-severe
Bocchino, 2022 2 84 0.02 [0.00; 0.08]
Bongiovanni, 2023 13 86 0.15 [0.08; 0.24]
Guo, 2022 19 146 0.13 [0.08; 0.20]
Huang, 2022 3 10 0.30 [0.07; 0.65]
Lerum, 2023 42 94 0.45 [0.34; 0.55]
Liao, 2022 20 98 0.20 [0.13; 0.30]
Liu, 2022 0 52 0.00 [0.00; 0.07]
Lorent, 2022 18 48 0.38 [0.24; 0.53]
Tarraso, 2022 40 99 0.40 [0.31; 0.51]
Zhang, 2022 11 192 0.06 [0.03; 0.10]
Zhao, 2021 18 51 0.35 [0.22; 0.50]
Zhou, 2021 11 83 0.13 [0.07; 0.22]
van Raaij, 2022 8 38 0.21 [0.10; 0.37]
van der Sar-van der Brugge, 2023 6 17 0.35 [0.14; 0.62]
Random effects model 1098 0.17 [0.10; 0.28]
Heterogeneity: I^2 = 87%, τ^2 = 1.1370, p < 0.01
Severity = severe
Bongiovanni, 2023 26 147 0.18 [0.12; 0.25]
Corsi, 2022 2 63 0.03 [0.00; 0.11]
Eberst, 2022 32 64 0.50 [0.37; 0.63]
Faverio, 2021 61 270 0.23 [0.18; 0.28]
Flor, 2023 1 18 0.06 [0.00; 0.27]
Gamberini, 2021 21 37 0.57 [0.39; 0.73]
González, 2022 20 41 0.49 [0.33; 0.65]
Guo, 2022 9 62 0.15 [0.07; 0.26]
Han, 2021 7 62 0.11 [0.05; 0.22]
Huang, 2022 31 47 0.66 [0.51; 0.79]
Lerum, 2023 21 32 0.66 [0.47; 0.81]
Liao, 2022 43 158 0.27 [0.20; 0.35]
Liu, 2022 0 434 0.00 [0.00; 0.01]
Lorent, 2022 21 57 0.37 [0.24; 0.51]
Martino, 2022 7 47 0.15 [0.06; 0.28]
Noureddine, 2023 29 60 0.48 [0.35; 0.62]
Núñez-Fernández, 2023 13 70 0.19 [0.10; 0.30]
Pan, 2022 50 209 0.24 [0.18; 0.30]
Schlemmer, 2023 73 123 0.59 [0.50; 0.68]
Tarraso, 2022 31 57 0.54 [0.41; 0.68]
Wu, 2021 19 83 0.23 [0.14; 0.33]
Zhang, 2022 0 7 0.00 [0.00; 0.41]
Zhao, 2021 20 43 0.47 [0.31; 0.62]
Zhou, 2021 5 14 0.36 [0.13; 0.65]
van Raaij, 2022 11 26 0.42 [0.23; 0.63]
van der Sar-van der Brugge, 2023 25 49 0.51 [0.36; 0.66]
Random effects model 2280 0.26 [0.17; 0.38]
Heterogeneity: I^2 = 88%, τ^2 = 1.9028, p < 0.01
0 0.2 0.4 0.6 0.8

Reticulation

1-6 Months

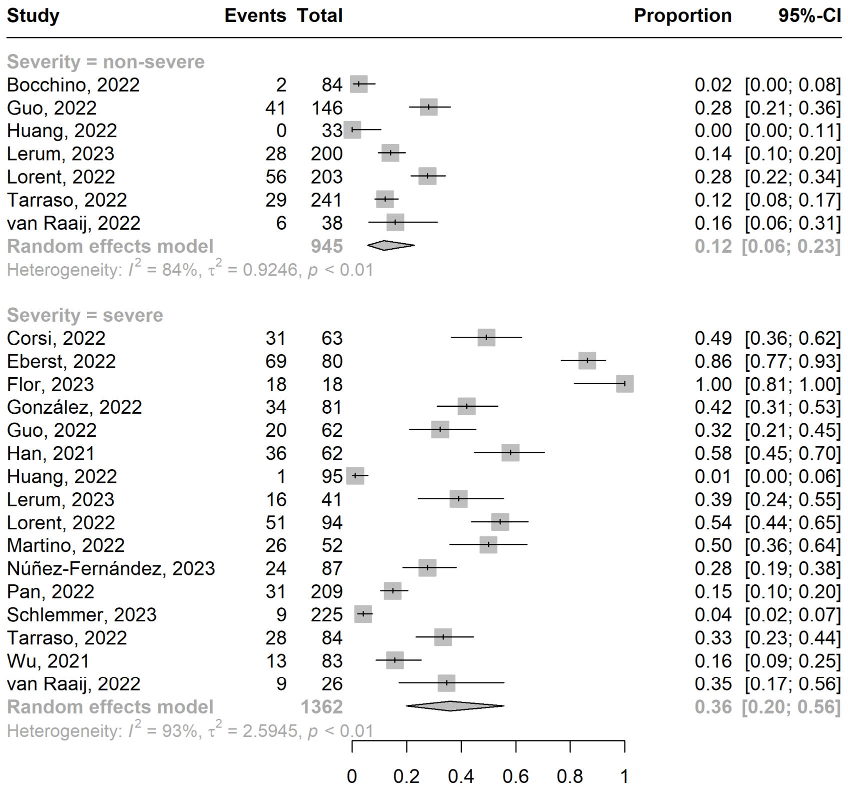

12-24 Months

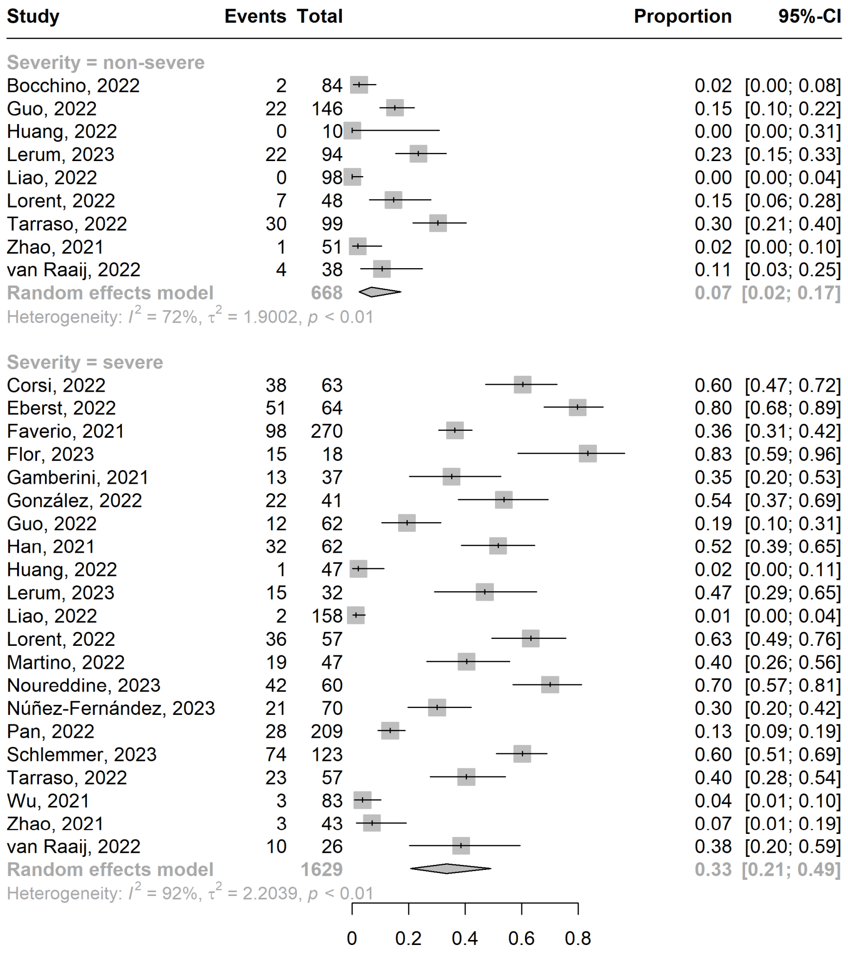
Study Events Total Proportion 95%-CI
Severity = non-severe
Bocchino, 2022 2 84 0.02 [0.00; 0.08]
Guo, 2022 22 146 0.15 [0.10; 0.22]
Huang, 2022 0 10 0.00 [0.00; 0.31]
Lerum, 2023 22 94 0.23 [0.15; 0.33]
Liao, 2022 0 98 0.00 [0.00; 0.04]
Lorent, 2022 7 48 0.15 [0.06; 0.28]
Tarraso, 2022 30 99 0.30 [0.21; 0.40]
Zhao, 2021 1 51 0.02 [0.00; 0.10]
van Raaij, 2022 4 38 0.11 [0.03; 0.25]
Random effects model 668 0.07 [0.02; 0.17]
Heterogeneity: I^2 = 72%, τ^2 = 1.9002, p < 0.01
Severity = severe
Corsi, 2022 38 63 0.60 [0.47; 0.72]
Eberst, 2022 51 64 0.80 [0.68; 0.89]
Faverio, 2021 98 270 0.36 [0.31; 0.42]
Flor, 2023 15 18 0.83 [0.59; 0.96]
Gamberini, 2021 13 37 0.35 [0.20; 0.53]
González, 2022 22 41 0.54 [0.37; 0.69]
Guo, 2022 12 62 0.19 [0.10; 0.31]
Han, 2021 32 62 0.52 [0.39; 0.65]
Huang, 2022 1 47 0.02 [0.00; 0.11]
Lerum, 2023 15 32 0.47 [0.29; 0.65]
Liao, 2022 2 158 0.01 [0.00; 0.04]
Lorent, 2022 36 57 0.63 [0.49; 0.76]
Martino, 2022 19 47 0.40 [0.26; 0.56]
Noureddine, 2023 42 60 0.70 [0.57; 0.81]
Núñez-Fernández, 2023 21 70 0.30 [0.20; 0.42]
Pan, 2022 28 209 0.13 [0.09; 0.19]
Schlemmer, 2023 74 123 0.60 [0.51; 0.69]
Tarraso, 2022 23 57 0.40 [0.28; 0.54]
Wu, 2021 3 83 0.04 [0.01; 0.10]
Zhao, 2021 3 43 0.07 [0.01; 0.19]
van Raaij, 2022 10 26 0.38 [0.20; 0.59]
Random effects model 1629 0.33 [0.21; 0.49]
Heterogeneity: I^2 = 92%, τ^2 = 2.2039, p < 0.01
0 0.2 0.4 0.6 0.8

Consolidation

1-6 Months

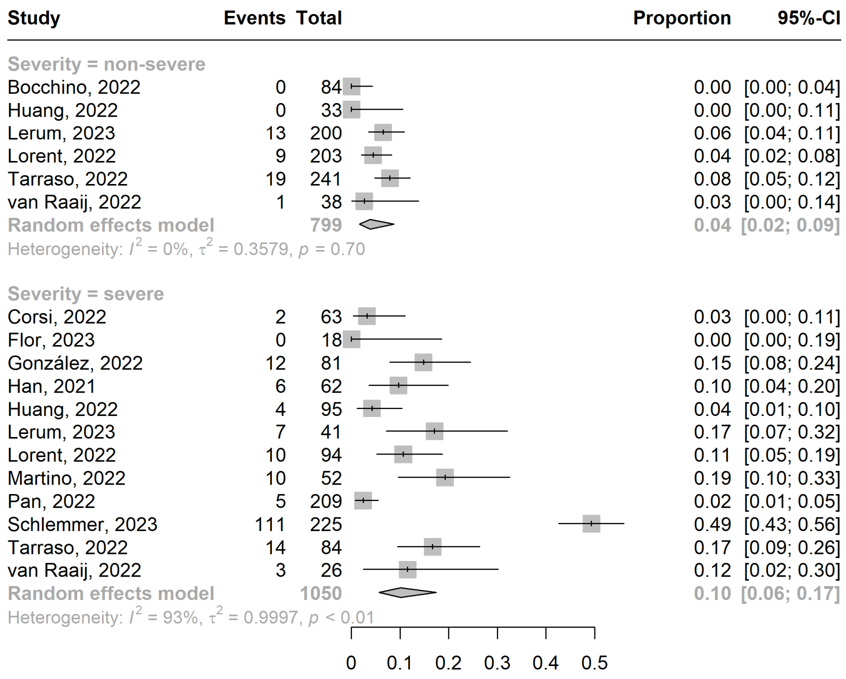

12-24 Months

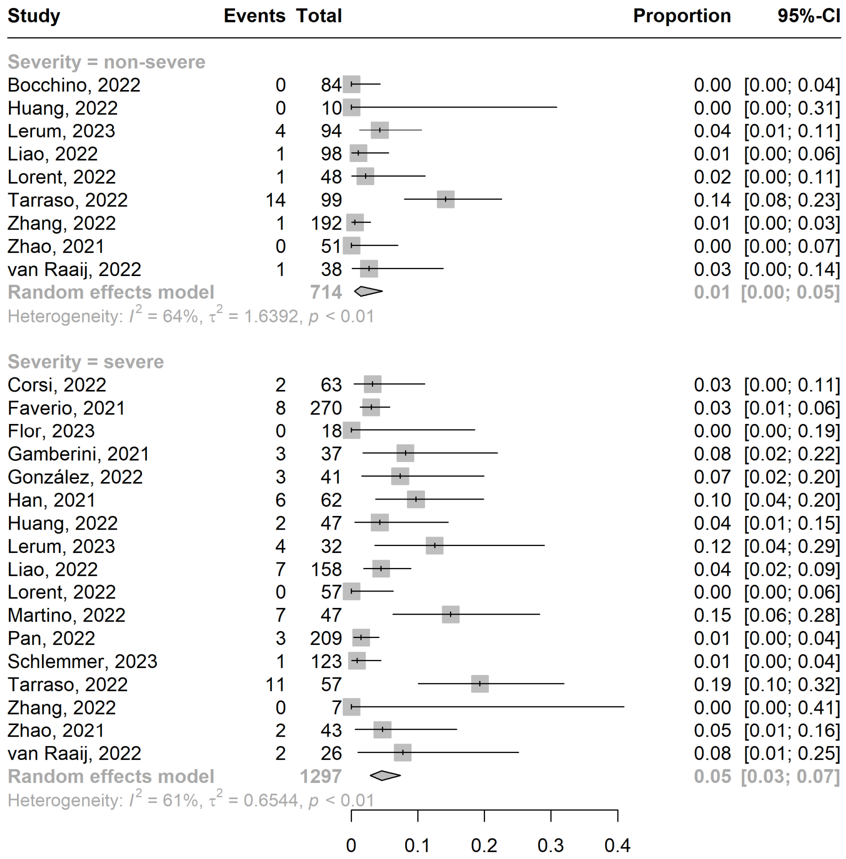

Interlobular Septal Thickening

1-6 Months

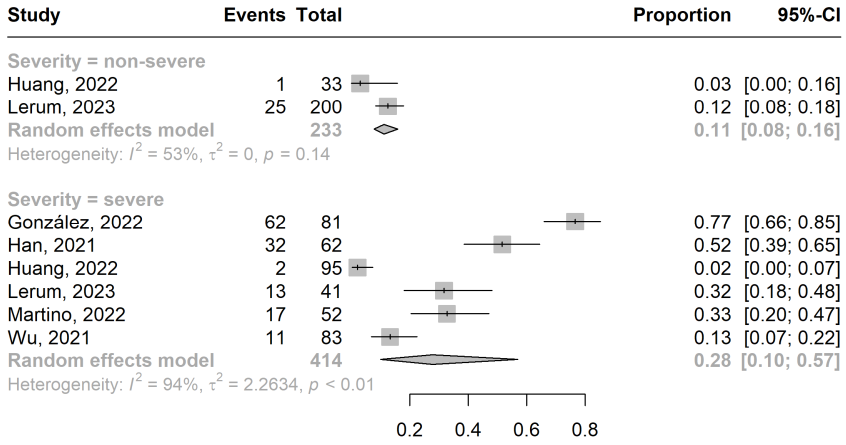

12-24 Months

Study	Events	Total	Proportion	95%-CI
**Severity = non-severe**				
Huang, 2022	0	10	0.00	[0.00; 0.31]
Lerum, 2023	9	94	0.10	[0.04; 0.17]
Zhang, 2022	13	192	0.07	[0.04; 0.11]
Zhao, 2021	3	51	0.06	[0.01; 0.16]
**Random effects model**		**347**	**0.07**	**[0.05; 0.10]**
Heterogeneity: $I^2 = 0\%$, $\tau^2 = 0$, $p = 0.82$				
**Severity = severe**				
González, 2022	41	41	1.00	[0.91; 1.00]
Han, 2021	28	62	0.45	[0.32; 0.58]
Huang, 2022	4	47	0.09	[0.02; 0.20]
Lerum, 2023	8	32	0.25	[0.11; 0.43]
Martino, 2022	5	47	0.11	[0.04; 0.23]
Wu, 2021	4	83	0.05	[0.01; 0.12]
Zhang, 2022	0	7	0.00	[0.00; 0.41]
Zhao, 2021	7	43	0.16	[0.07; 0.31]
**Random effects model**		**362**	**0.22**	**[0.05; 0.63]**
Heterogeneity: $I^2 = 81\%$, $\tau^2 = 5.8363$, $p < 0.01$				

Honeycombing

1-6 Months

Study	Events	Total	Proportion	95%-CI
**Severity = non-severe**				
Bocchino, 2022	0	84	0.00	[0.00; 0.04]
**Severity = severe**				
Eberst, 2022	6	80	0.07	[0.03; 0.16]
Flor, 2023	2	18	0.11	[0.01; 0.35]
Martino, 2022	3	52	0.06	[0.01; 0.16]
Schlemmer, 2023	11	225	0.05	[0.02; 0.09]
**Random effects model**		**375**	**0.06**	**[0.04; 0.09]**
Heterogeneity: $I^2 = 0\%$, $\tau^2 = 0$, $p = 0.66$				

12-24 Months

Study	Events	Total	Proportion	95%-CI
**Severity = non-severe**				
Bocchino, 2022	0	84	0.00	[0.00; 0.04]
**Severity = severe**				
Eberst, 2022	3	64	0.05	[0.01; 0.13]
Faverio, 2021	3	270	0.01	[0.00; 0.03]
Flor, 2023	2	18	0.11	[0.01; 0.35]
Gamberini, 2021	3	37	0.08	[0.02; 0.22]
Martino, 2022	2	47	0.04	[0.01; 0.15]
Noureddine, 2023	3	60	0.05	[0.01; 0.14]
Schlemmer, 2023	13	123	0.11	[0.06; 0.17]
**Random effects model**		**619**	**0.05**	**[0.03; 0.09]**
Heterogeneity: $I^2 = 60\%$, $\tau^2 = 0.4090$, $p = 0.02$				

Supplemental Figure S4. Meta-Regression Plots for the Pooled Event Rate Each Lung Abnormality Stratified by Primary COVID-19 Infection Severity Using Months Since Primary Infection as the Independent Predictor

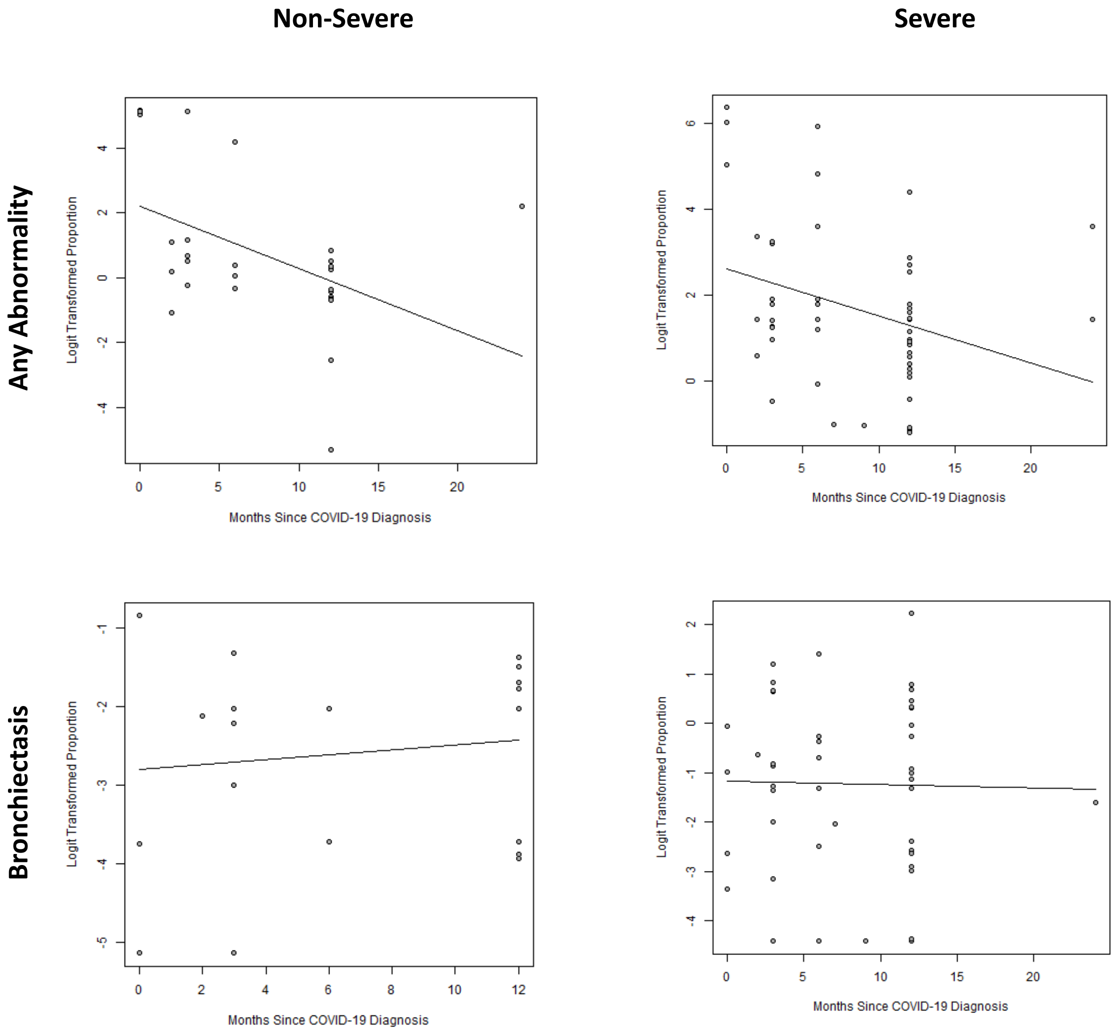

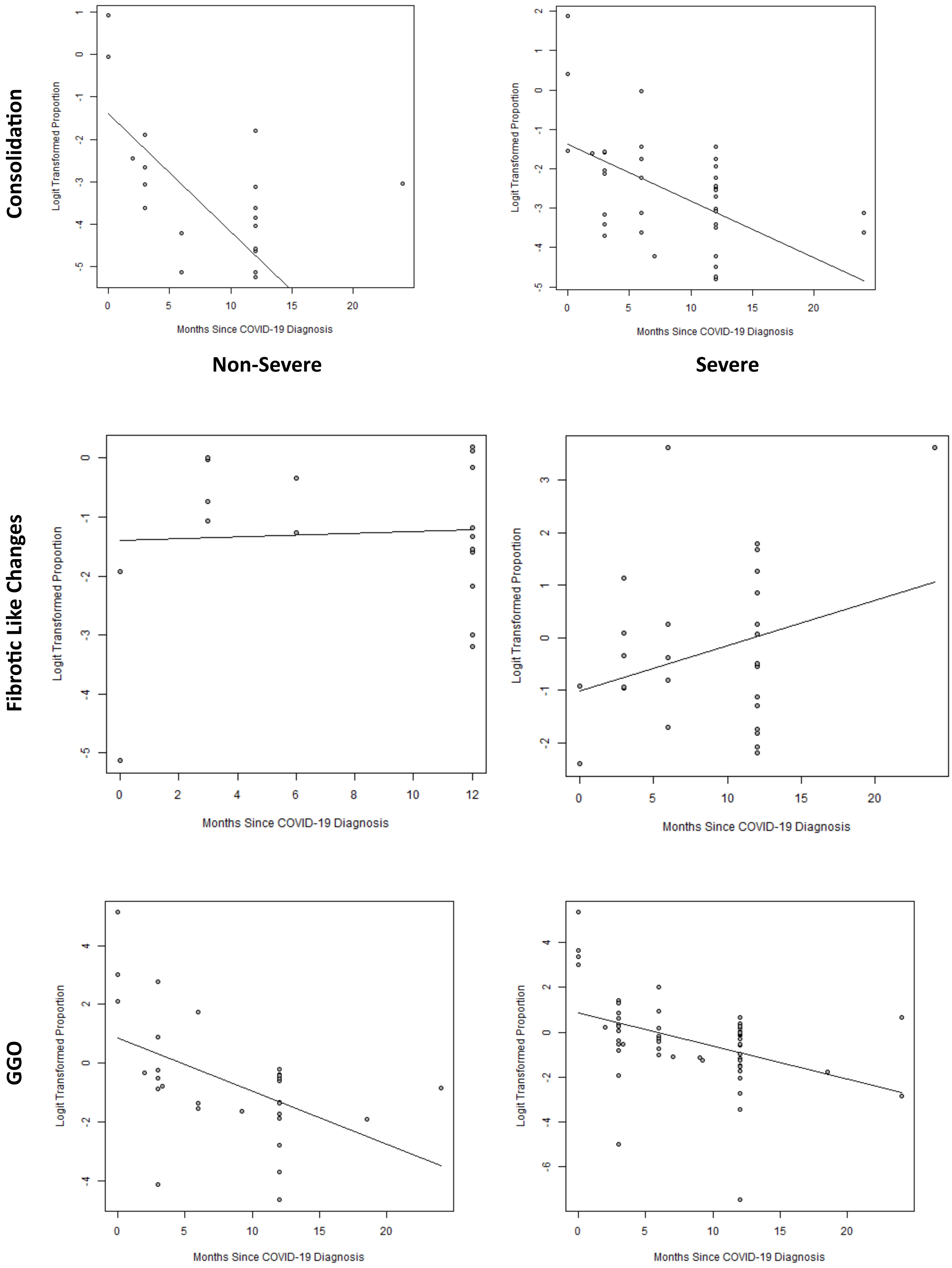
Consolidation
Fibrotic Like Changes
GGO
Non-Severe
Severe
Logit Transformed Proportion
Months Since COVID-19 Diagnosis

Honeycombing

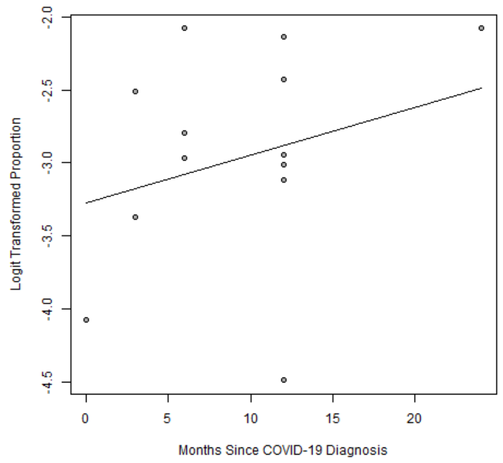

Non-Severe **Severe**

Interlobular Septal Thickening

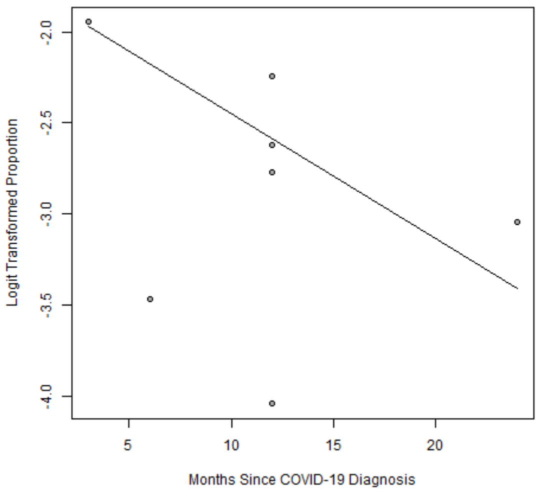

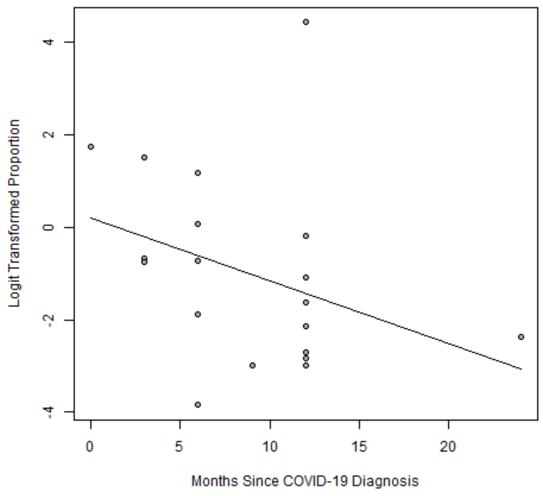

Reticulation

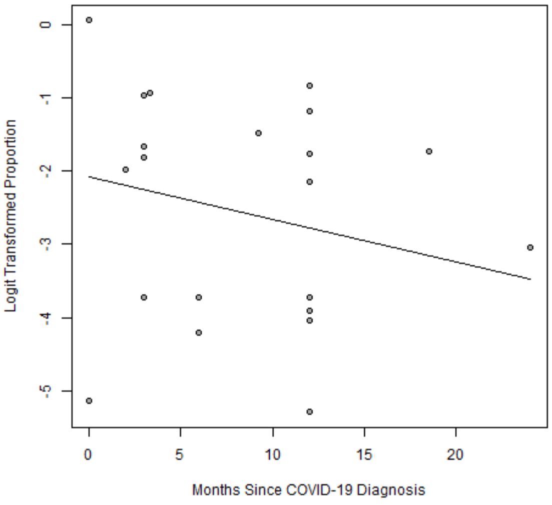

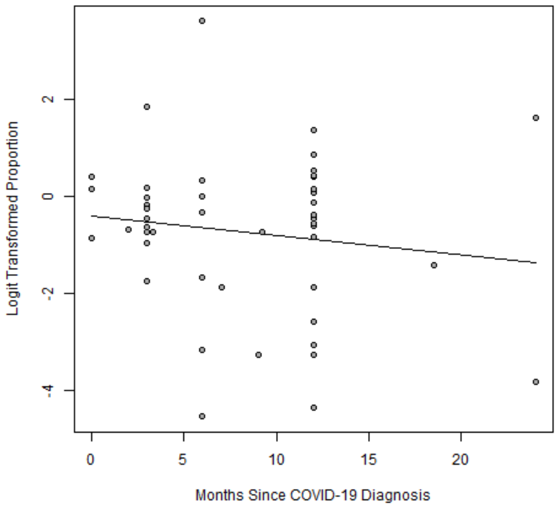

Supplemental Figure S5. Risk of Bias Assessment Summary Plot Using Hoy et al. Tool

The tool consists of 10 items addressing four domains of bias. Items 1 to 4 assess the external validity of the study (domains are selection and nonresponse bias), and items 5 to 10 assess the internal validity (items 5 to 9 assess the domain of measurement bias, and item 10 assesses bias related to the analysis).

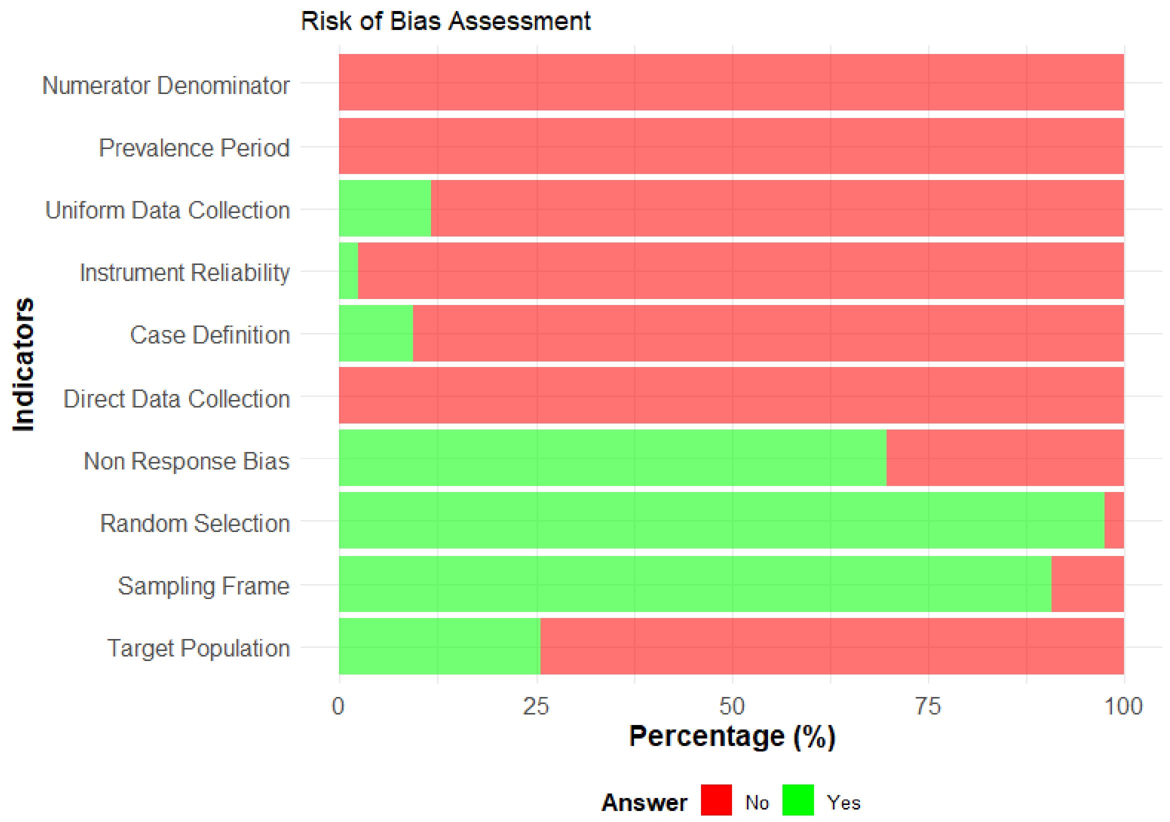

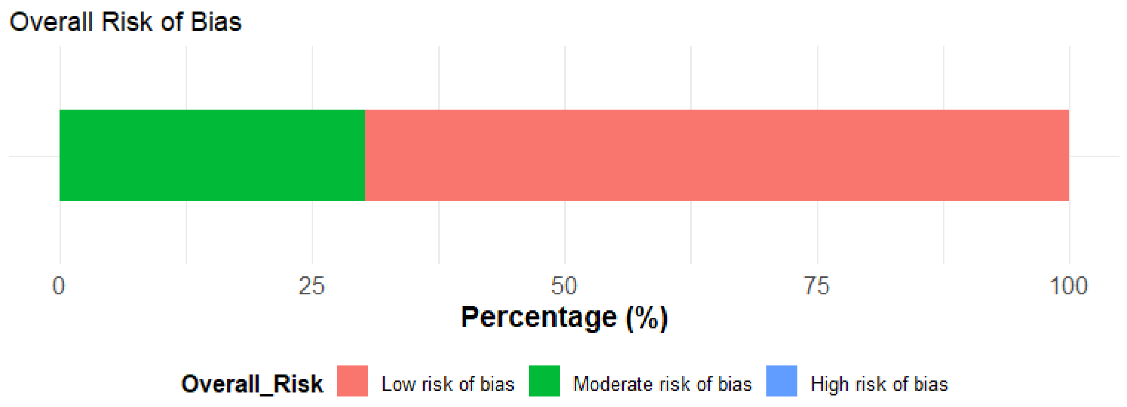

Supplemental Figure S6. Funnel Plots for the Pooled Meta-Analysis

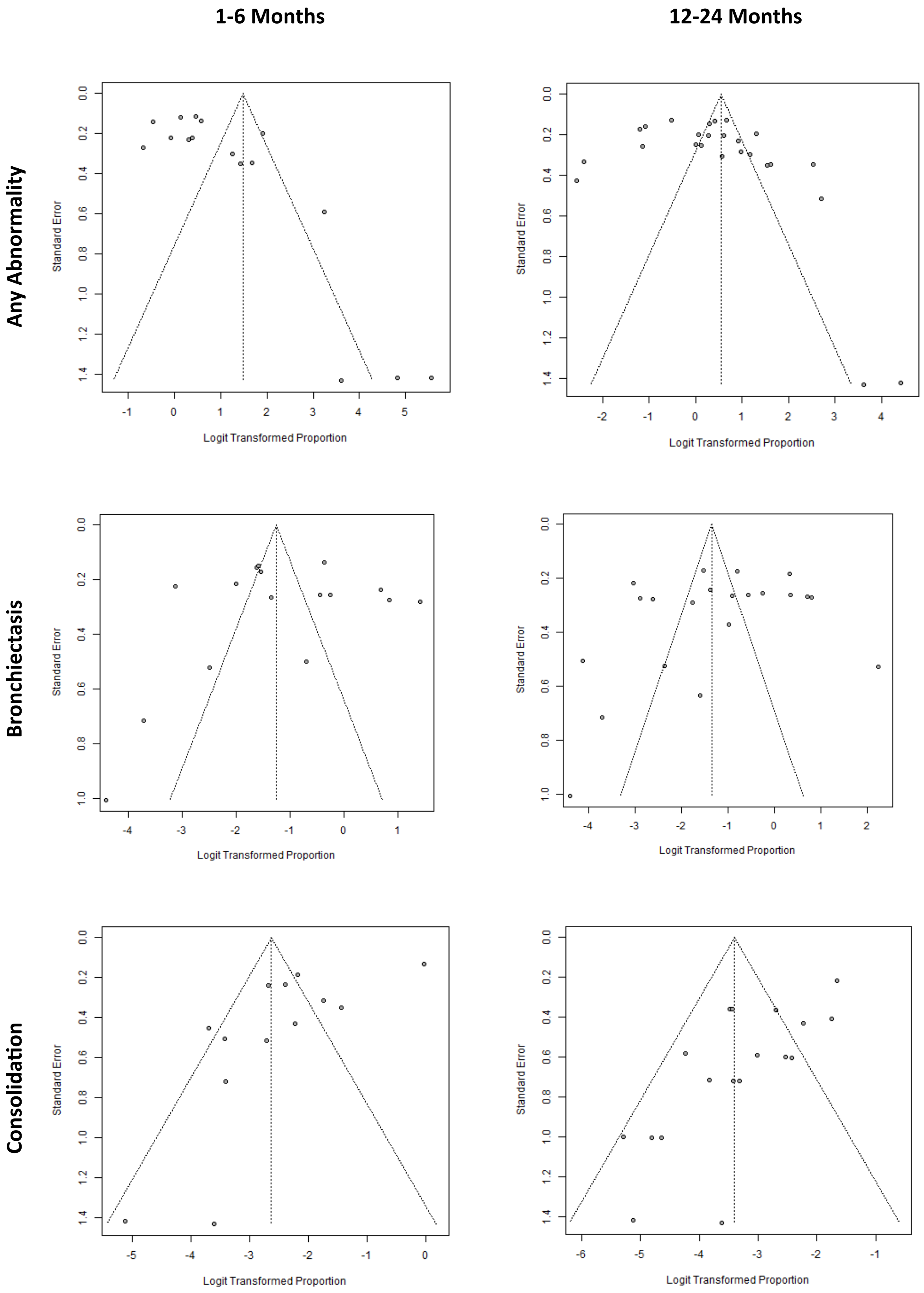

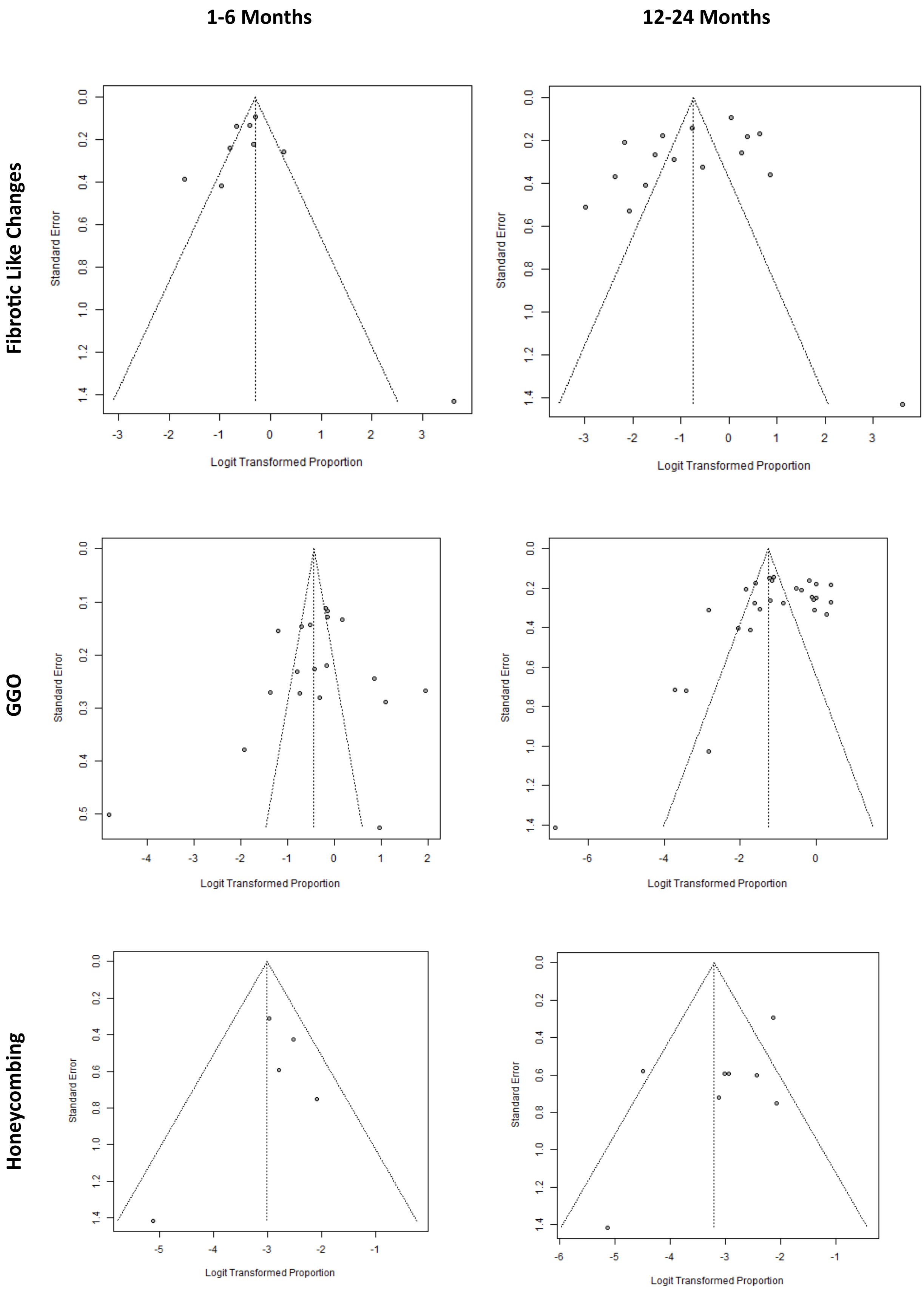
1-6 Months
12-24 Months
Fibrotic Like Changes
GGO
Honeycombing
Standard Error
Logit Transformed Proportion

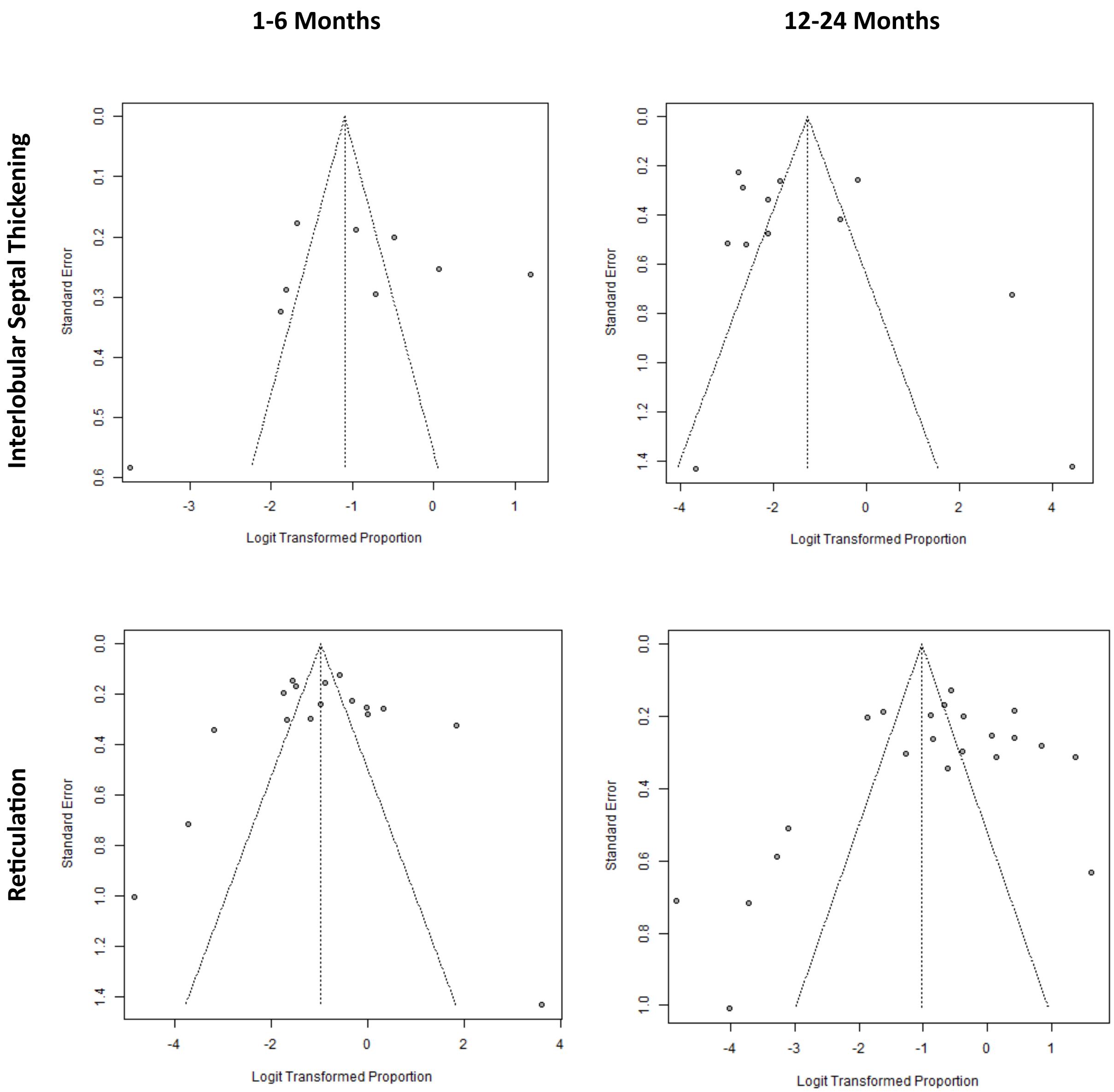
1-6 Months
12-24 Months
Interlobular Septal Thickening
Reticulation
Standard Error
Logit Transformed Proportion

Supplemental Figure S7. Funnel Plots for the Stratified Meta-Analysis by Severity

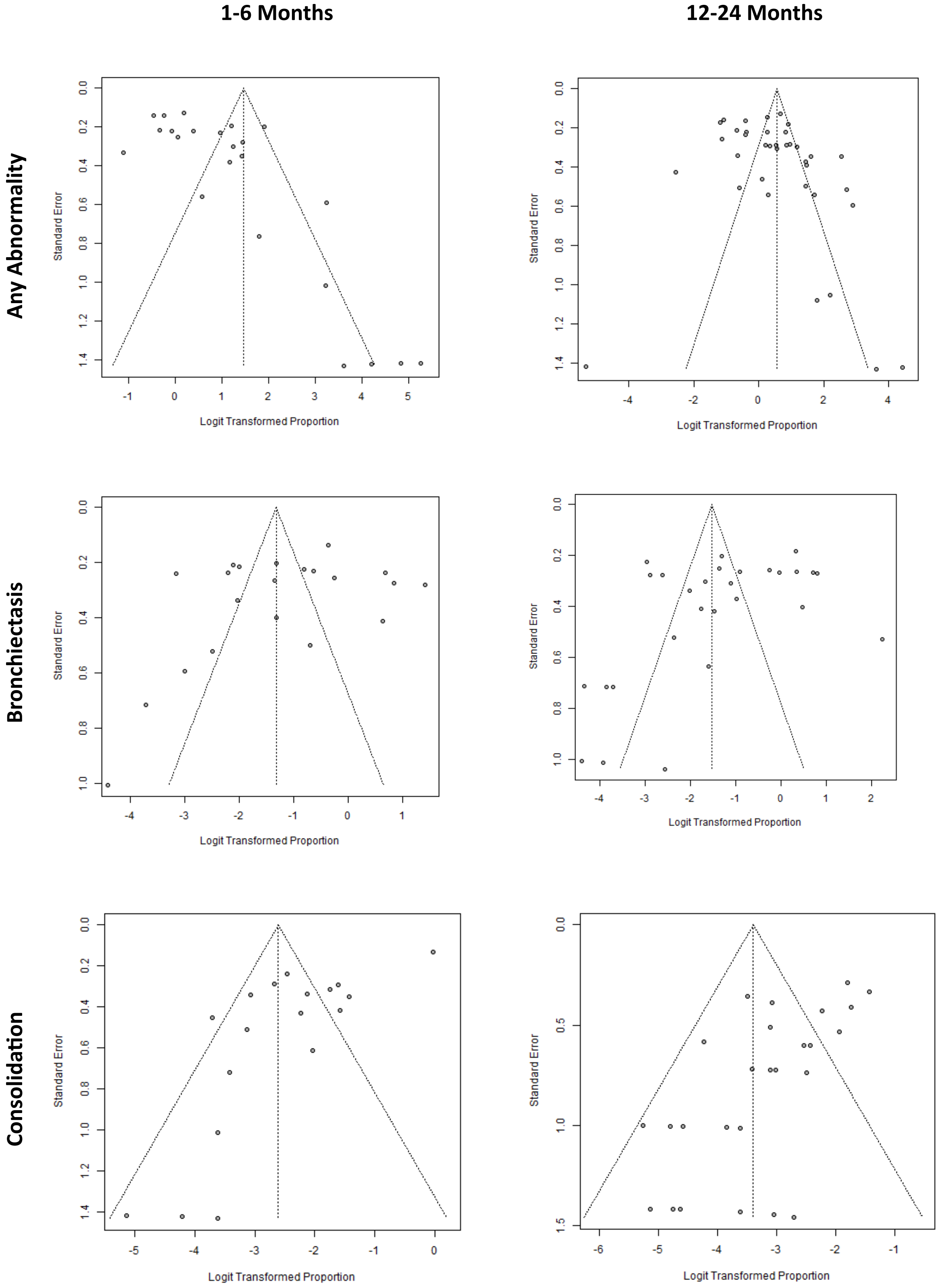

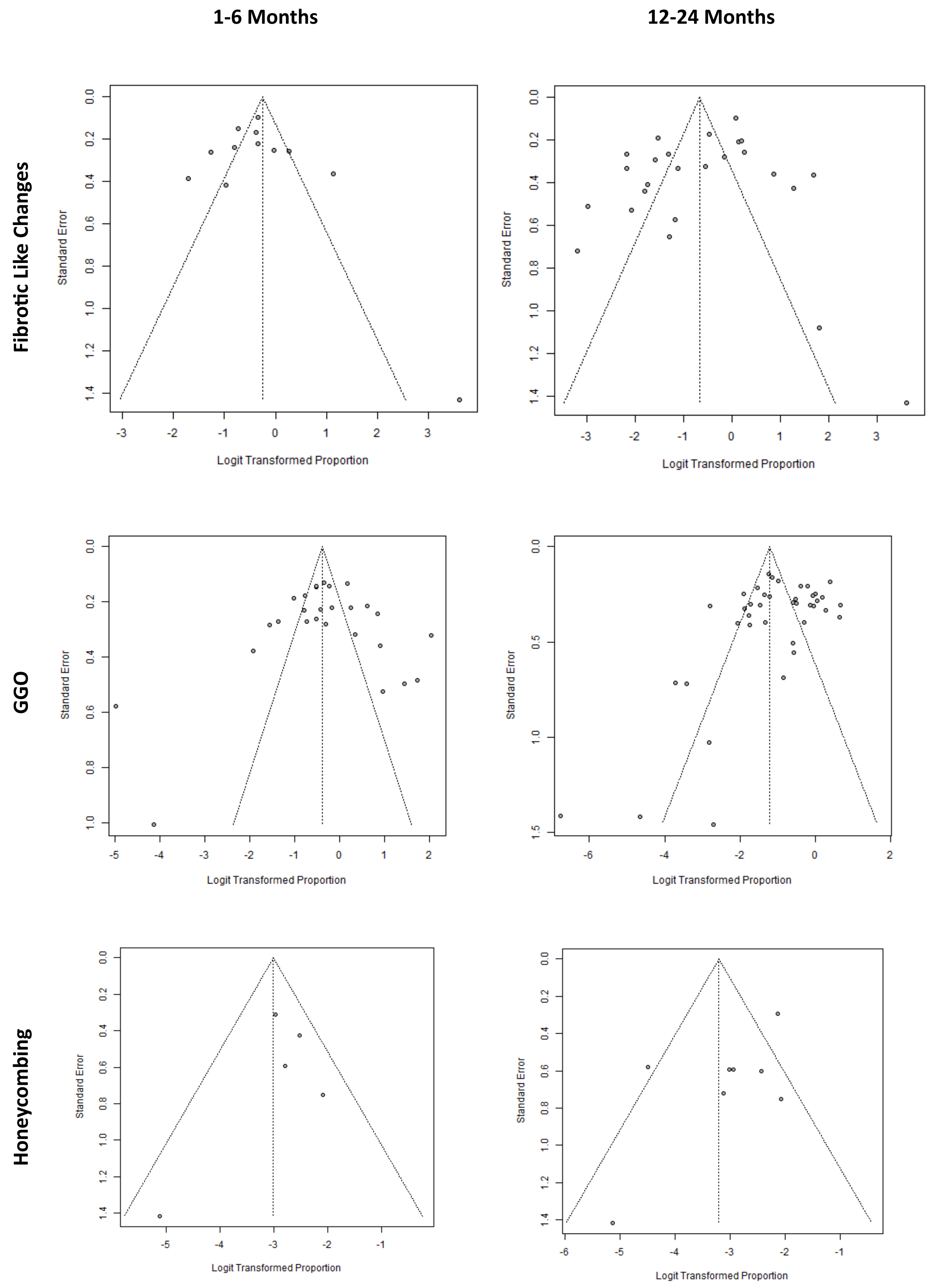
1-6 Months
12-24 Months
Fibrotic Like Changes
GGO
Honeycombing
Standard Error
Logit Transformed Proportion

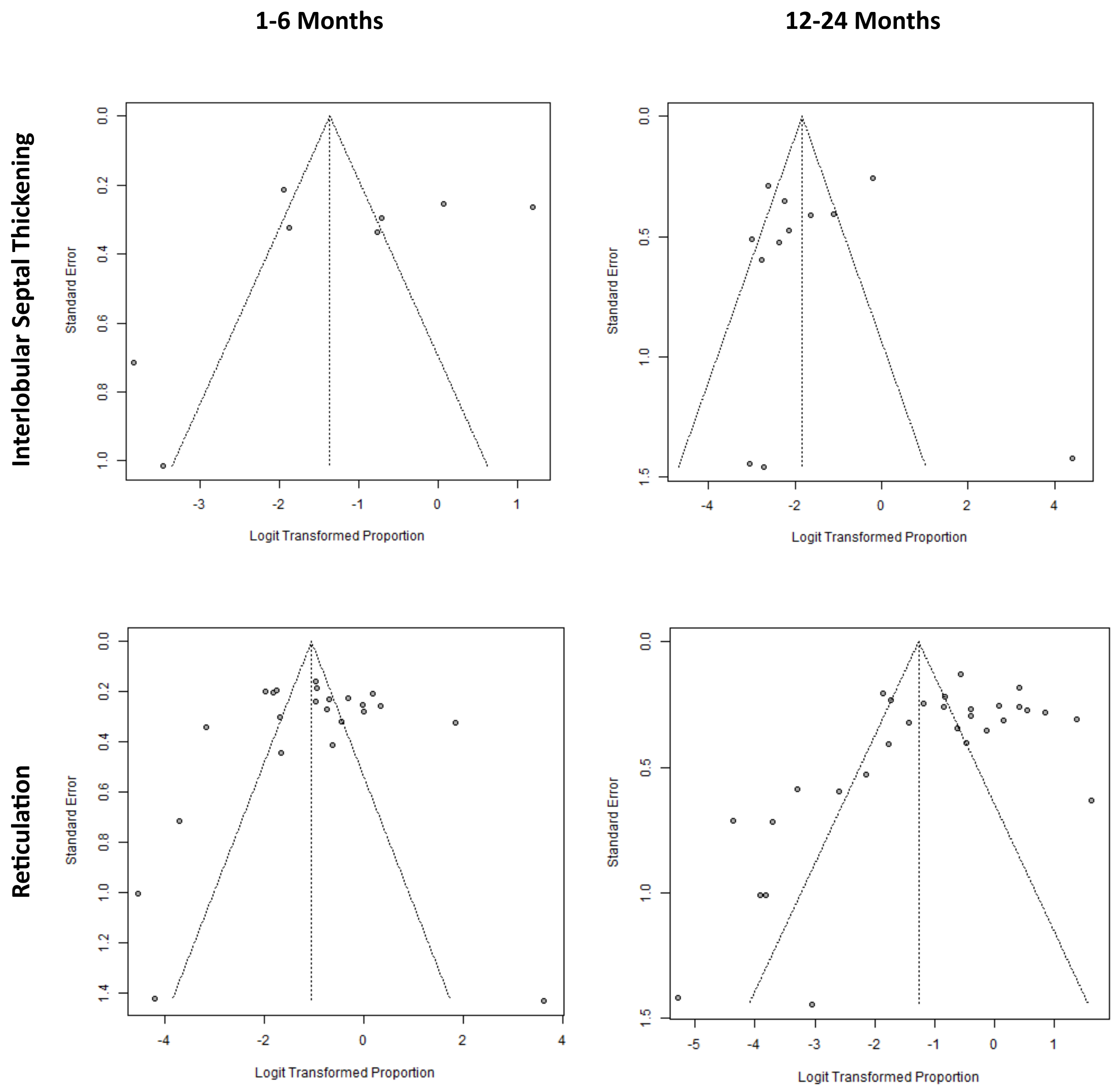
1-6 Months
12-24 Months
Interlobular Septal Thickening
Reticulation
Standard Error
Standard Error
Standard Error
Standard Error
Logit Transformed Proportion
Logit Transformed Proportion
Logit Transformed Proportion
Logit Transformed Proportion
